# Synthesis, characterization, aggregation-induced emission, solvatochromism and mechanochromism of fluorinated benzothiadiazole bonded to tetraphenylethenes†

**DOI:** 10.1039/c8ra01448e

**Published:** 2018-04-03

**Authors:** Chin-Yang Yu, Chia-Chieh Hsu, Hsi-Chen Weng

**Affiliations:** Department of Materials Science and Engineering, National Taiwan University of Science and Technology 43, Section 4, Keelung Road Taipei 10607 Taiwan cyyu@mail.ntust.edu.tw

## Abstract

Compounds consisting of unsubstituted, monofluoro and difluoro substituted benzothiadiazole bonded to two tetraphenylethenes were successfully prepared by palladium catalyzed Suzuki–Miyaura cross-coupling reaction of their corresponding co-monomers. All compounds exhibited aggregation-induced emission characteristics when the water fraction was higher than 60% in the THF/water mixtures. The emission maximum for the three compounds was blue-shifted when the water content reached 90% compared to that in THF solution. The intensity of emission maximum of difluorinated benzothiadiazole linked with two tetraphenylethenes was 2.5 times higher in 90% water compared to those in THF solution. Surprisingly, two liquid crystal phases with two distinct emission colors were observed only for the compound containing difluorinated benzothiadiazole bonded to two tetraphenylethene. All compounds showed remarkable solvatochromic properties in selected solvents with different polarities. The powder XRD results and mechanochromism of the compounds suggested that the solid state structures can change from one form to another by grinding, fuming or annealing processes.

## Abstract 2

Fluorinated benzothiadiazole bonded to two tetraphenylethenes were synthesized. The compounds exhibited remarkable aggregation-induced emission, solvatochromism and mechanochromism.

## Graphical abstract

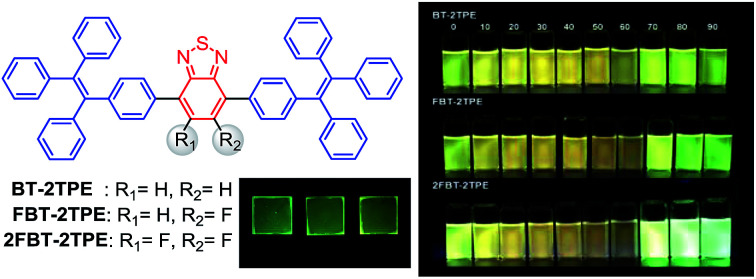

## Introduction

Fluorescent organic molecules have attracted much attention on account of their outstanding sensitivity and distinct selectivity^1,2^ and have been widely used for the detection of a variety of analytes in the solution state.^3–5^ Considering their potential use in optoelectronic devices such as organic light-emitting diodes (OLEDs), organic light-emitting field-effect transistors (OLEFETs) and organic solid lasers, the molecules must exhibit high emission intensity in the solid state. However, conventional fluorescent molecules exhibit relatively low fluorescence in the solid state due to the strong aggregation caused quenching effect.^6–8^ Recently, the aggregation induced emission (AIE) characteristics of small molecules and polymers has been proposed.^9–12^ Tetraphenylethene (TPE)^13–15^ with its propeller-shaped structure is one of the most studied examples for aggregation induced emission characteristics. Very recently, the TPE based molecules have been synthesized and investigated by Misra *et al.*^16–18^ The rational molecular design of the TPE based molecules makes it potential uses in non-doped OLEDs with a good color contrast. In addition, the obvious color change ranging from blue to red with reversible mechanochromism based on molecules containing the TPE linked to a variety of electron acceptors has also been demonstrated.

Typically, the emission colors of conjugated carbocyclic compounds with AIE characteristics are between blue and green.^19^ In fact, the emission colors of compounds can be altered through molecular design such as when the structures contain electron donor–acceptor (D–A) units and heteroatoms. In addition, the rational design and synthesis of D–A molecules could realize full visible colors by manipulating the strength of intramolecular charge transfer (ICT) between the constituent units.^20^ Benzothiadiazole (BT) is one of the most important classes of electron acceptors due to its relatively high electron affinity and π-extended planar structure.^21^ Recently, fluorination of BT has been utilized and shown to be an efficient means to modify the optical, electronic and electrochemical properties of BT.^22,23^ Tang and coworkers have synthesized the electron donor–acceptor–donor (D–A–D) chromophores containing BT derivatives and TPE.^24^ These molecules exhibit high emission with different emission colors in the solid state. The other asymmetrical D–A or D–A–D types of chromophores such as cyano-substituted diphenylethene derivatives with AIE characteristics, vapochromic and mechanochromic properties have also been reported.^25–27^ The compounds revealed a reversible conversion between the crystalline and amorphous forms by different processes.

Solvatochromism is the ability of compounds to change color when dissolved in different solvents.^28,29^ Recently, a variety of solvatochromic molecules with D–A structures including symmetrical or asymmetrical benzothiadiazide based cyano-substituted diphenylethene derivatives^26,30,31^ have been synthesized and studied due to their applicability as probes for the determination of volatile solvent vapor as well as their potential application in optical recording and security papers. These compounds exhibit remarkable solvatochromic and vapochromic characteristics by conjugation effects, molecular packing and conversion of crystalline to amorphous states. Mechanofluorochromic materials are a sort of smart material which display a changing color of emission in response to external stimuli and these materials can be potentially used in memory chips, security ink and sensors.^32–34^ Araki and coworkers have reported that organic mechanofluorochromic compounds exhibited piezochromic luminescence by controlling the mode of molecular packing.^35^ Mechanofluorochromism of donor–acceptor π-conjugated (D–π–A) fluorescent dyes was attributed to a reversible switching between crystalline and amorphous states with a change in the dipole–dipole and intermolecular π–π interactions.^36,37^ Secondary bonding interactions and D–A pairs play important roles in the design of new mechanochromic materials.^38–40^ Recently, a number of D–A compounds have been prepared and found to possess mechanofluorochromic nature.^41–43^

In general, D–A type molecular systems show low fluorescence quantum efficiency in solution as well as in the solid state. Although the AIE active molecules containing BT bonded to two TPE with remarkable mechanochromism have been reported and investigated by Misra,^44^ no examples of fluorinated substituted BT bonded to two TPE are available. The modification of the fluorine atoms onto the BT unit seems to be an efficient way that the molecules can be easily prepared and the optical, electronic and electrochemical properties as well as aggregation-induced emission characteristics, solvatochromism and mechanochromism can be fine-tuned which can compare to that of the unsubstituted BT bonded to two TPE. The aim of this research work is mainly to investigate the properties and the effect of fluorine atoms attached to the central BT unit of D–A–D type molecular systems as well as their aggregation-induced emission characteristics, solvatochromism and mechanochromism. In this work, we report the synthesis of unsubstituted, monofluoro and difluoro substituted BT bonded to two TPE by palladium catalyzed Suzuki–Miyaura cross-coupling reaction of their corresponding comonomers. The structures of the D–A–D types of three chromophores have been fully characterized. Aggregation induced emission characteristics of small molecules in the THF/water mixtures and in the solid state have been studied as well as their solvatochromic and mechanochromic effects related to their structures.

## Results and discussion

### Synthesis and characterization of BT-2TPE, FBT-2TPE and 2FBT-2TPE

**Scheme 1:**
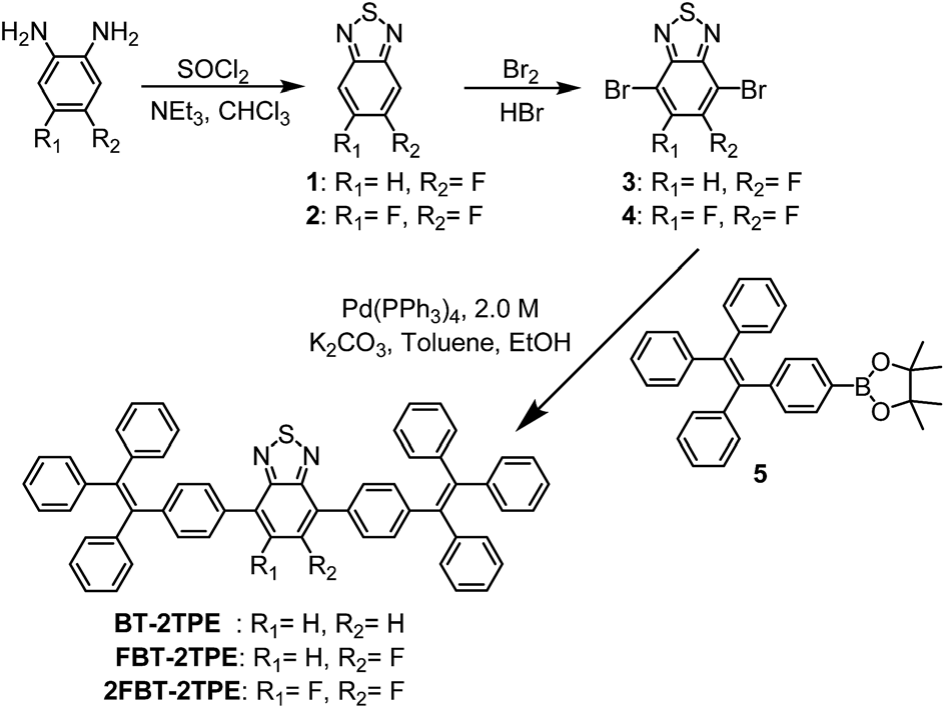
Synthetic routes to BT-2TPE, FBT-2TPE and 2FBT-2TPE.

The synthetic routes towards the D–A–D types of molecules BT-2TPE, FBT-2TPE and 2FBT-2TPE are shown in Scheme 1. Fluorinated phenylenediamine derivatives were reacted with thionyl chloride (SOCl_2_) in the presence of triethylamine in anhydrous chloroform to afford the fluorinated 2,1,3-benzothiadiazole derivatives 1 and 2. A high selectivity of bromination at the 4 and 7 position of 1 and 2 was carried out by the dropwise addition of bromine in hydrobromic acid containing 1 or 2 to give 3 and 4. The 4-(1,2,2-triphenylvinyl)phenylboronic acid pinacol ester 5 was prepared by a modification of a procedure reported previously.^45^ The D–A–D types of molecules BT-2TPE, FBT-2TPE and 2FBT-2TPE were synthesized by Suzuki–Miyaura cross-coupling reaction using equimolar amounts of the corresponding comonomers, 10 mol% Pd(PPh_3_)_4_ and aqueous K_2_CO_3_ to give BT-2TPE, FBT-2TPE and 2FBT-2TPE in a yield of 85%, 64% and 38%, respectively.

The compounds BT-2TPE, FBT-2TPE and 2FBT-2TPE were fully characterized by ^1^H NMR, ^13^C NMR, ^19^F NMR, ^1^H–^1^H COSY spectroscopy and high resolution mass spectrometry (see ESI†). Clearly, a low isolated yield of compounds FBT-2TPE and 2FBT-2TPE was obtained compared to that of BT-2TPE possibly due to the low reactivity of the fluorinated BT moieties as the introduction of the fluorine atom to the BT leads to a deactivation of the aromatic ring.

**Fig. 1:**
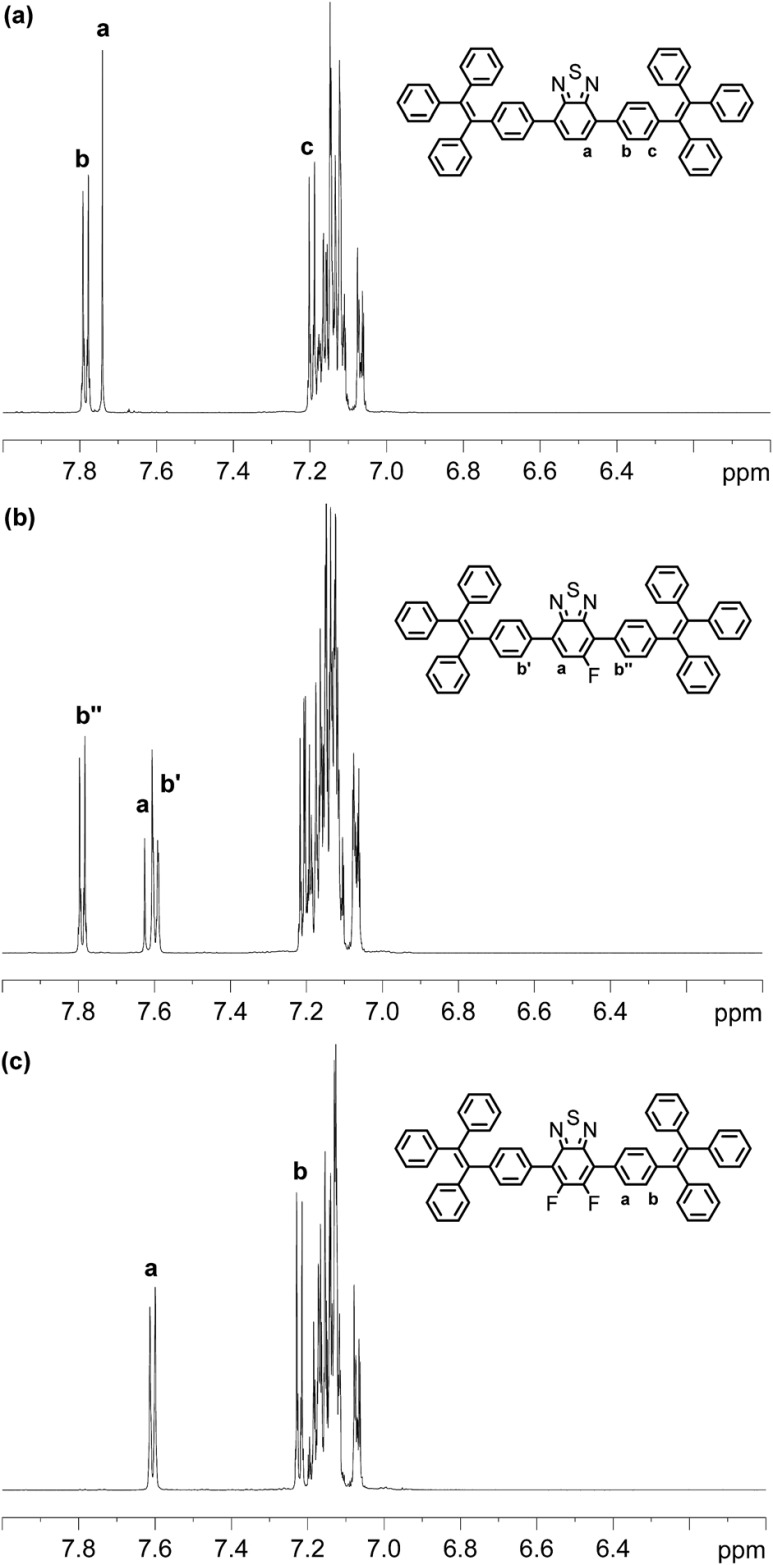
^1^H NMR spectra of BT-2TPE, FBT-2TPE and 2FBT-2TPE in CD_2_Cl_2_.

Compounds BT-2TPE, FBT-2TPE and 2FBT-2TPE were characterized by ^1^H NMR spectroscopy in CD_2_Cl_2_ (Fig. 1). A singlet peak at 7.74 ppm integrating to 2 hydrogens corresponds to the hydrogens of BT unit for BT-2TPE (Fig. 1a). Two AA′BB′ systems at around 7.78 and 7.20 ppm integrating to 4 hydrogens each were assigned to the hydrogens of TPE relative to *ortho* and *meta* position of carbons linked to the BT moieties, respectively. The multiple signals between 7.05 and 7.18 ppm integrating to 30 hydrogens were attributed to the remaining hydrogens of the TPE. The doublet at around 7.63 ppm (*J*_H–F_ = 11.8 Hz) integrating to 1 hydrogen was assigned to the hydrogen of BT unit relative to the *ortho* position of carbon linked to fluorine atom in FBT-2TPE (Fig. 1b). The two AA′BB′ systems at 7.79 and 7.60 ppm integrating to 2 hydrogens each were attributed to the hydrogens of phenyl ring of TPE moieties relative to the *ortho* position of carbon linked to BT units. The multiple signals between 7.04 and 7.23 ppm integrating to 34 hydrogens were attributed to the remaining hydrogens of the TPE. The multiple signals appearing at 7.61 and 7.22 ppm (two AA′BB′ systems) integrating to 4 hydrogens each correspond to the hydrogens of the phenyl ring relative to the *ortho* and the *meta* position of carbons attached to the difluoro-substituted BT, respectively (Fig. 1c). Again, the multiple signals between 7.05 and 7.20 ppm integrating to 30 hydrogens are attributed to the remaining hydrogens of the TPE.

### Optical properties of BT-2TPE, FBT-2TPE and 2FBT-2TPE

**Fig. 2:**
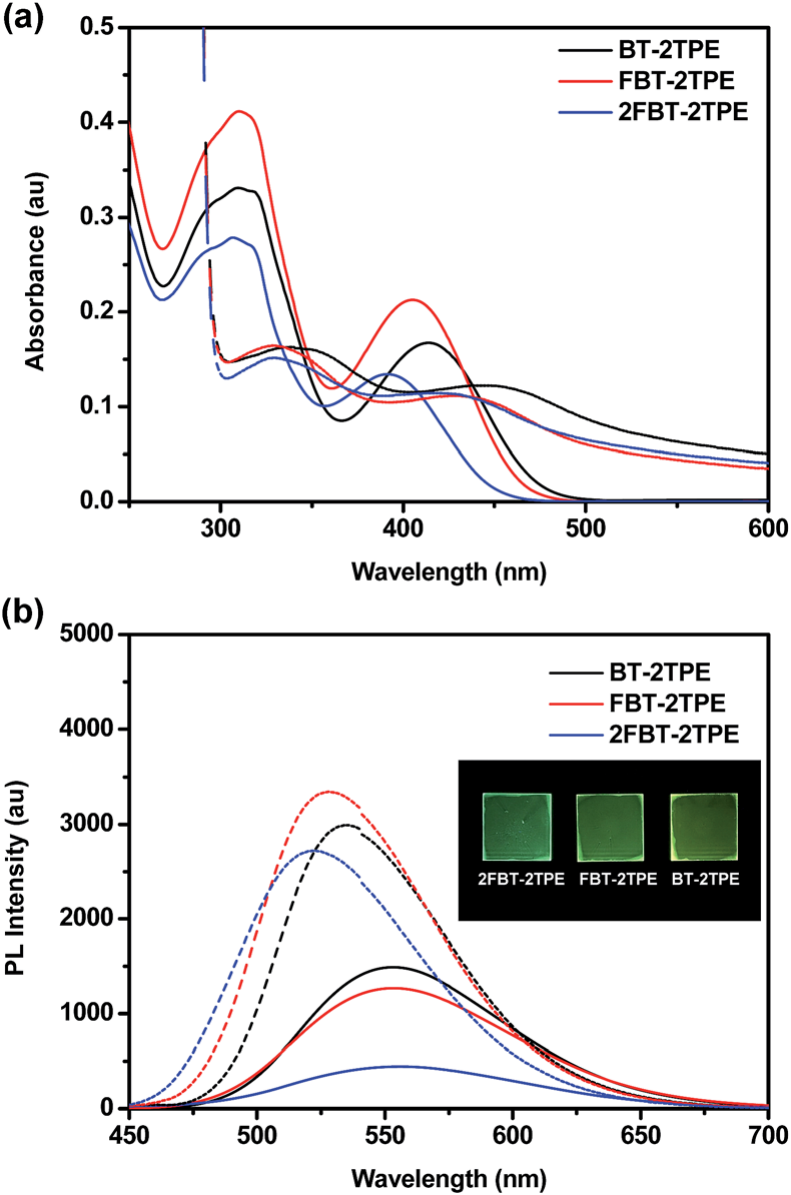
(a) UV-Vis spectra and (b) emission spectra of BT-2TPE, FBT-2TPE and 2FBT-2TPE in dichloromethane solution (solid line) and in solid state (dashed line). Pictures of films under UV light irradiation at a wavelength of 365 nm are shown in the insets.

The UV-vis absorption spectra of BT-2TPE, FBT-2TPE and 2FBT-2TPE were recorded in dilute dichloromethane (5 × 10^−6^ M) (Fig. 2a). The band between 280 and 350 nm can be attributed to the π–π* transition and another band between 360 and 470 nm corresponds to the charge transfer (CT) transition between the TPE and the BT units.^46^ The absorption maximum of BT-2TPE, FBT-2TPE and 2FBT-2TPE is at 414, 405 and 391 nm, respectively. The absorption maximum was blue-shifted after the introduction of the fluorine atom onto the BT possibly due to a collapsing of the planarity of the molecules. The emission maximum wavelength of BT-2TPE, FBT-2TPE and 2FBT-2TPE in dilute dichloromethane (5 × 10^−6^ M) (Fig. 2b) is very similar at around 553 nm. Thin films of BT-2TPE, FBT-2TPE and 2FBT-2TPE were prepared by spin-coating 150 μL of the solution (3 mg mL^−1^ in toluene) onto glass slides (2.0 cm × 2.0 cm) at a rotation speed of 1500 rpm for 20 seconds and then 3000 rpm for 20 seconds. The solid state absorption maximum of BT-2TPE, FBT-2TPE and 2FBT-2TPE was red-shifted by 30, 24 and 26 nm, respectively compared to that of the solution. This bathochromic shift can be attributed to intermolecular interactions in the solid state as we expected. However, the solid state emission maximum wavelength of BT-2TPE, FBT-2TPE and 2FBT-2TPE is blue-shifted by 18, 26 and 38 nm compared to that of the solution. The optical bandgap calculated by the onset of the absorption edge of BT-2TPE, FBT-2TPE and 2FBT-2TPE is 2.35, 2.40 and 2.46 eV, respectively. The introduction of a fluorine atom onto the BT units leads to a collapse of the planarity of the molecules, therefore the optical bandgap increases as we expected. The details of the absorption and emission maximum, quantum yield, Stokes shift and optical bandgap are shown in the ESI (Table S1†).

**Fig. 3:**
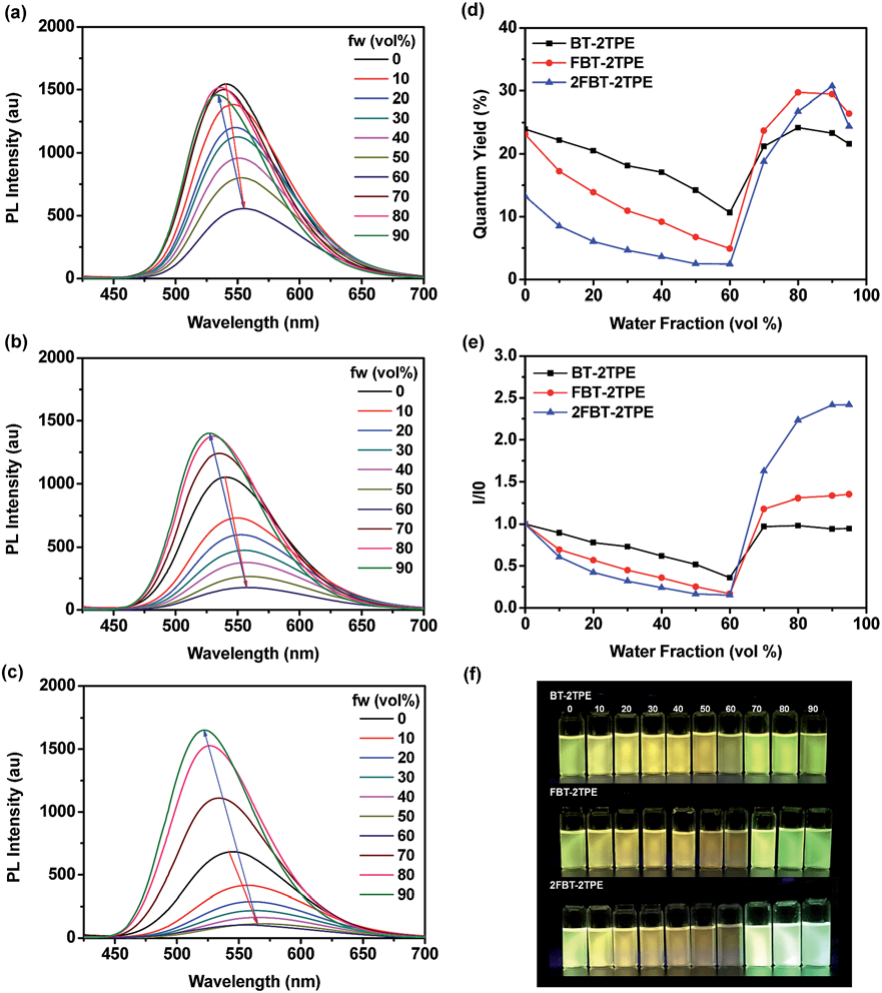
Emission spectra of (a) BT-2TPE, (b) FBT-2TPE and (c) 2FBT-2TPE in THF-water mixtures (concentration:5 μM; excitation wavelength: *λ*_max_) and plots of (d) fluorescence quantum yields and (e) *I*/*I*_0_*vs.* water fraction of BT-2TPE, FBT-2TPE and 2FBT-2TPE and (f) pictures of BT-2TPE, FBT-2TPE and 2FBT-2TPE in different THF-water mixtures under UV light irradiation at a wavelength of 365 nm.

The emission spectra of compounds BT-2TPE, FBT-2TPE and 2FBT-2TPE with different water fractions in THF/water mixture are shown in Fig. 3a–c. Upon increasing the water fraction up to 60%, the maximum emission wavelength of BT-2TPE, FBT-2TPE and 2FBT-2TPE was red-shifted by 15, 18 and 23 nm, respectively and the maximum emission intensity gradually decreases. This could be attributed to the stabilization of the intramolecular charge-transfer state upon increasing the polarity of the solvents.^46,47^ However, upon a further increase in the water fraction from 60% to 90%, the maximum emission wavelength of BT-2TPE, FBT-2TPE and 2FBT-2TPE was blue-shifted and the maximum emission intensity generally increases due to formation of nanoaggregates that can enhance aggregation-induced emission. All compounds showed distinct responses in different THF and water percentages. At lower water fractions (<60%), the emission is mainly attributed to intramolecular charge transfer between the electron donor and the electron acceptor which is controlled by solvent polarity, however, at higher water contents (>60%), AIE dominates over solvent polarity. The maximum photoluminescence quantum yield of compounds BT-2TPE, FBT-2TPE and 2FBT-2TPE (Fig. 3d) is 0.24, 0.29 and 0.31, respectively compared to quinine sulfate (0.546) in 0.1 M H_2_SO_4_. It should be noted that the emission maximum intensity of 2FBT-2TPE (Fig. 3e) in 90% water content was 2.5 times higher than that of in THF solution. The pictures of BT-2TPE, FBT-2TPE and 2FBT-2TPE in different THF-water mixtures under UV light irradiation at a wavelength of 365 nm is shown in Fig. 3f. All compounds BT-2TPE, FBT-2TPE and 2FBT-2TPE showed emission color changes upon increases in the fraction of water.

### Single crystal structure of 2FBT-2TPE

**Fig. 4:**
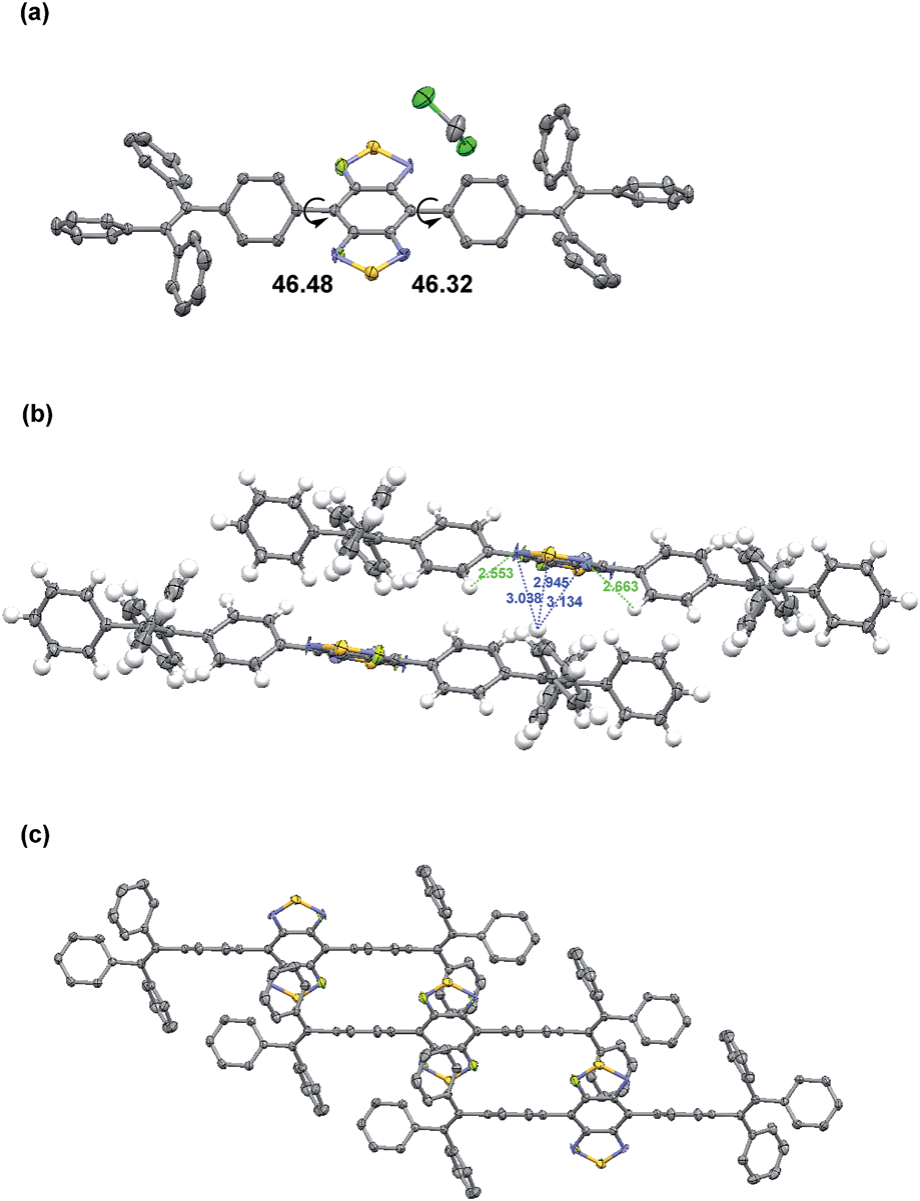
(a) Solid-state structures, (b) inter- and intramolecular distances and (c) packing diagram of 2FBT-2TPE. Thermal ellipsoids are set at 50% probability.

In order to obtain a single crystal of 2FBT-2TPE, a saturated dichloromethane solution was allowed to slowly evaporate at room temperature. The solid state structure of 2FBT-2TPE (Fig. 4a) showed symmetrical disorder in the BT core (space group: *P*1, *Z* = 1) and solvent packing in the crystal structure. The crystal structure of 2FBT-2TPE confirmed that the dihedral angles between BT and phenyl rings of TPE were 46.32° and 46.48°, respectively which are relatively large due to the highly twisted structure of 2FBT-2TPE. The packing diagram of 2FBT-2TPE (Fig. 4b) showed the intermolecular distance of C–H⋯S was 2.945 Å from the disordered sulfur atom, the intermolecular distance of C–H⋯F was 3.038 and 3.134 Å from the disordered fluorine atom and intramolecular distance of C–H⋯F was 2.553 and 2.663 Å. The packing structure of 2FBT-2TPE (Fig. 4c) exhibited a ladder-like packing structure with a distance of 4.811 and 4.349 Å. An attempt to acquire the single crystal of BT-2TPE and FBT-2TPE failed possibly due to the highly random nature of the unsubstituted and mono-fluorinated substituted BT.

### Thermal properties of BT-2TPE, FBT-2TPE and 2FBT-2TPE

**Fig. 5:**
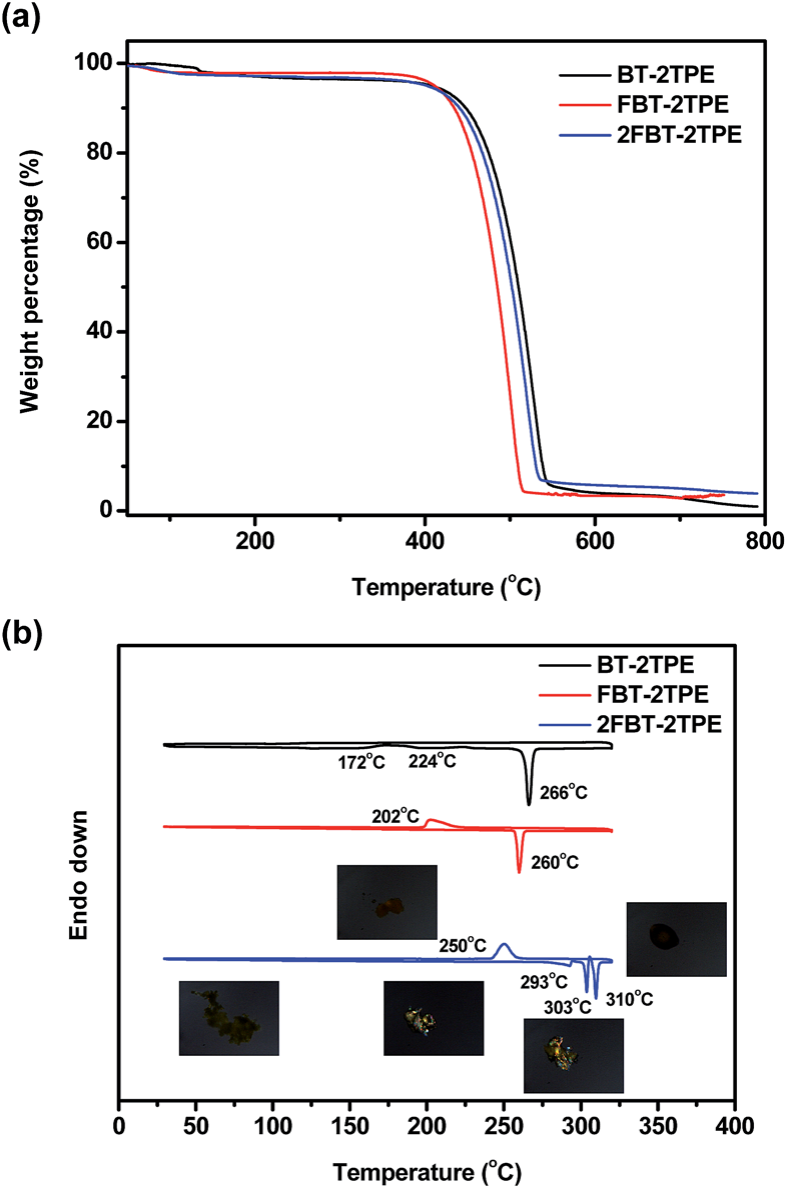
(a) TGA curves of BT-2TPE, FBT-2TPE and 2FBT-2TPE at a heating rate of 10 °C min^−1^ under a nitrogen atmosphere and (b) DSC diagrams of the second heating and cooling curves of BT-2TPE, FBT-2TPE and 2FBT-2TPE at a heating and cooling rate of 10 °C min^−1^ under a nitrogen atmosphere. Polarized optical micrographs associated with the transition temperature of 2FBT-2TPE are shown in the insets.

The decomposition temperatures of BT-2TPE, FBT-2TPE and 2FBT-2TPE were determined by thermal gravimetric analysis (TGA) at a heating rate of 10 °C min^−1^ under a nitrogen flow. Clearly, one step weight loss was observed for all compounds (Fig. 5a). The 5% weight loss of BT-2TPE, FBT-2TPE and 2FBT-2TPE was at 408 °C, 416 °C and 402 °C, respectively with a small amount of solid residue remaining when heating up to 800 °C. All compounds exhibited an excellent thermal stability. The DSC traces of BT-2TPE, FBT-2TPE and 2FBT-2TPE at the second heating cycle (Fig. 5b) showed an endothermic peak at 266, 260, 310 °C with an enthalpy value of 68.0, 46.6 and 17.5 kJ mol^−1^, respectively which can be attributed to the melting point of the compounds. In addition, two broad exothermic transitions for BT-2TPE at 172 and 224 °C were observed upon heating corresponding to the formation of a self-organized structure. The polarized optical micrographs associated with different transitions for 2FBT-2TPE were shown in the insets of Fig. 5b. There are an additional two endothermic peaks at 293 and 303 °C for 2FBT-2TPE which can be attributed to the two transitions related to the two distinct liquid crystal mesophases observed by polarized optical microscopy with a heating rate of 2 °Cmin^−1^. Upon cooling, the recrystallization temperature of FBT-2TPE and 2FBT-2TPE was observed at 202 and 250 °C, respectively. No recrystallization temperature for BT-2TPE could be observed possibly due to the random nature of BT-2TPE.

### Solvatochromic behaviors

**Fig. 6:**
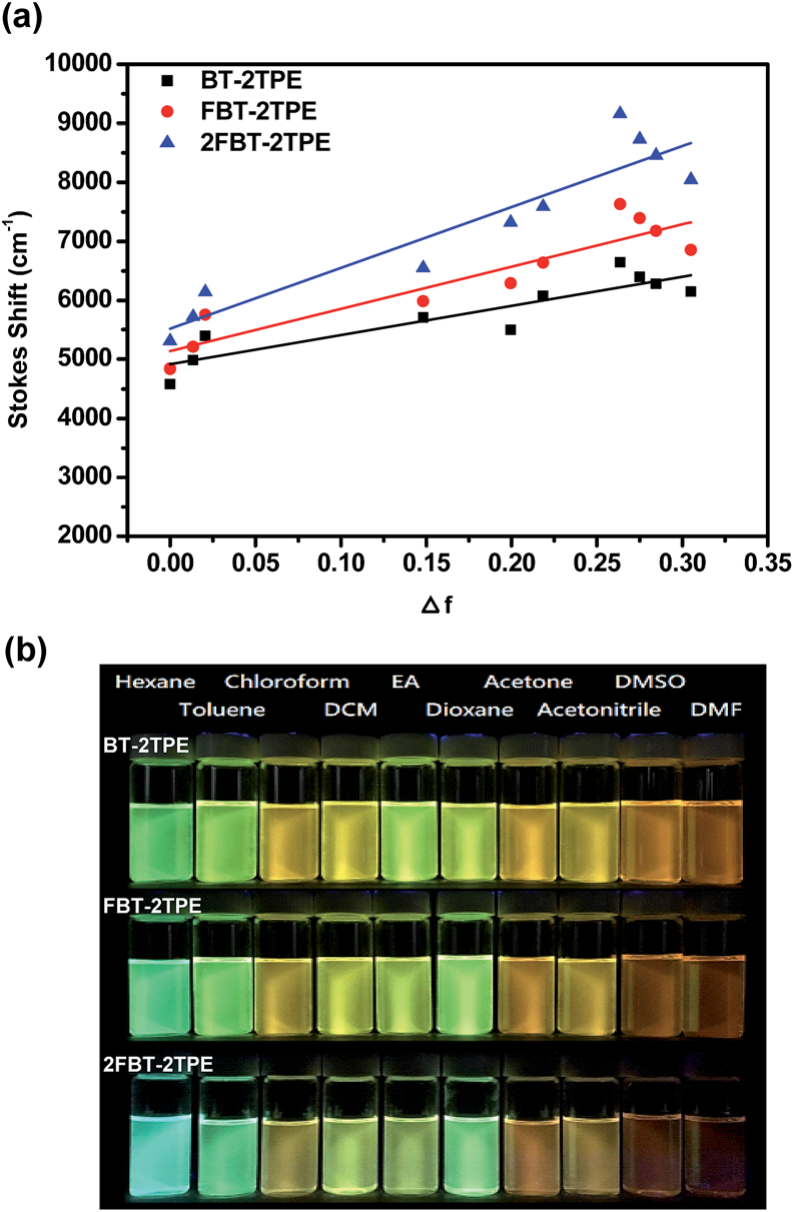
(a) Plot of Stokes shift *versus* solvent polarity parameter of BT-2TPE, FBT-2TPE and 2FBT-2TPE in various solvents and (b) pictures of BT-2TPE, FBT-2TPE, 2FBT-2TPE in different solvents under UV light at a wavelength of 365 nm.

In general, the spectral properties of D–A molecules are sensitive towards the solvent polarity. In order to study the solvatochromic effect of BT-2TPE, FBT-2TPE and 2FBT-2TPE, various solvents with different polarities such as hexane, toluene, chloroform, dichloromethane, ethyl acetate, 1,4-dioxane, acetone, acetonitrile, dimethylsufloxide and dimethylformaldehyde were selected. Compounds BT-2TPE, FBT-2TPE and 2FBT-2TPE were soluble in all of the selected organic solvents and exhibited a pronounced solvatochromic performance. Generally, the absorption maximum of BT-2TPE, FBT-2TPE and 2FBT-2TPE were all slightly blue-shifted when dissolved in a solvent of increasing polarity while the fluorescence maximum of BT-2TPE, FBT-2TPE and 2FBT-2TPE revealed a high degree of red-shifting with increasing solvent polarity. The emission maximum intensity gradually decreased, indicating that the larger charge separation and dipole moment existed in the excited state than in the ground state. The absorption spectra and emission spectra of BT-2TPE, FBT-2TPE and 2FBT-2TPE in different solvents and the solvatochromic behavior data have been summarized in the ESI (Fig. S32 and Table S3†). The plots of Stokes shift *versus* solvent polarity parameter based on the Lipper-Mataga equation of BT-2TPE, FBT-2TPE and 2FBT-2TPE were shown in Fig. 6a. As the solvent polarity parameter increases, the Stokes shift also increases with a linear correlation of 0.799 for BT-2TPE, 0.841 for FBT-2TPE and 0.863 for 2FBT-2TPE. In addition, the calculated slope of fitting line for BT-2TPE, FBT-2TPE and 2FBT-2TPE was 4954, 7169 and 10 334, respectively. These results indicate that the fluorescent properties of three compounds are strongly related to the solvent polarity. Compound 2FBT-2TPE exhibited a relatively steep slope indicating the formation of large charge separation and high dipole moment in the excited state. Pictures of BT-2TPE, FBT-2TPE and 2FBT-2TPE in different solvents under UV light at a wavelength of 365 nm were shown in Fig. 6b. A notable color change of three compounds in different polarities of solvents was observed indicating a remarkable solvatochromism.

### Mechanochromic behaviors

The ground samples were obtained from pristine powders *via* the grinding process using spatula, mortar and pestle. The annealed samples were obtained upon heating pristine powders at the specified temperature for 20 min and then allowing to slowly cool to room temperature. In this case, the annealing temperature for BT-2TPE, FBT-2TPE and 2FBT-2TPE was 200 °C, 200 °C and 270 °C, respectively. Surprisingly, the pristine powders BT-TPE obtained by evaporating dichloromethane and hexane exhibited a yellow and yellow-green color, respectively. The pristine powders FBT-2TPE and 2FBT-2TPE obtained by evaporating dichloromethane and hexane exhibited no color change upon visual inspection.

**Fig. 7:**
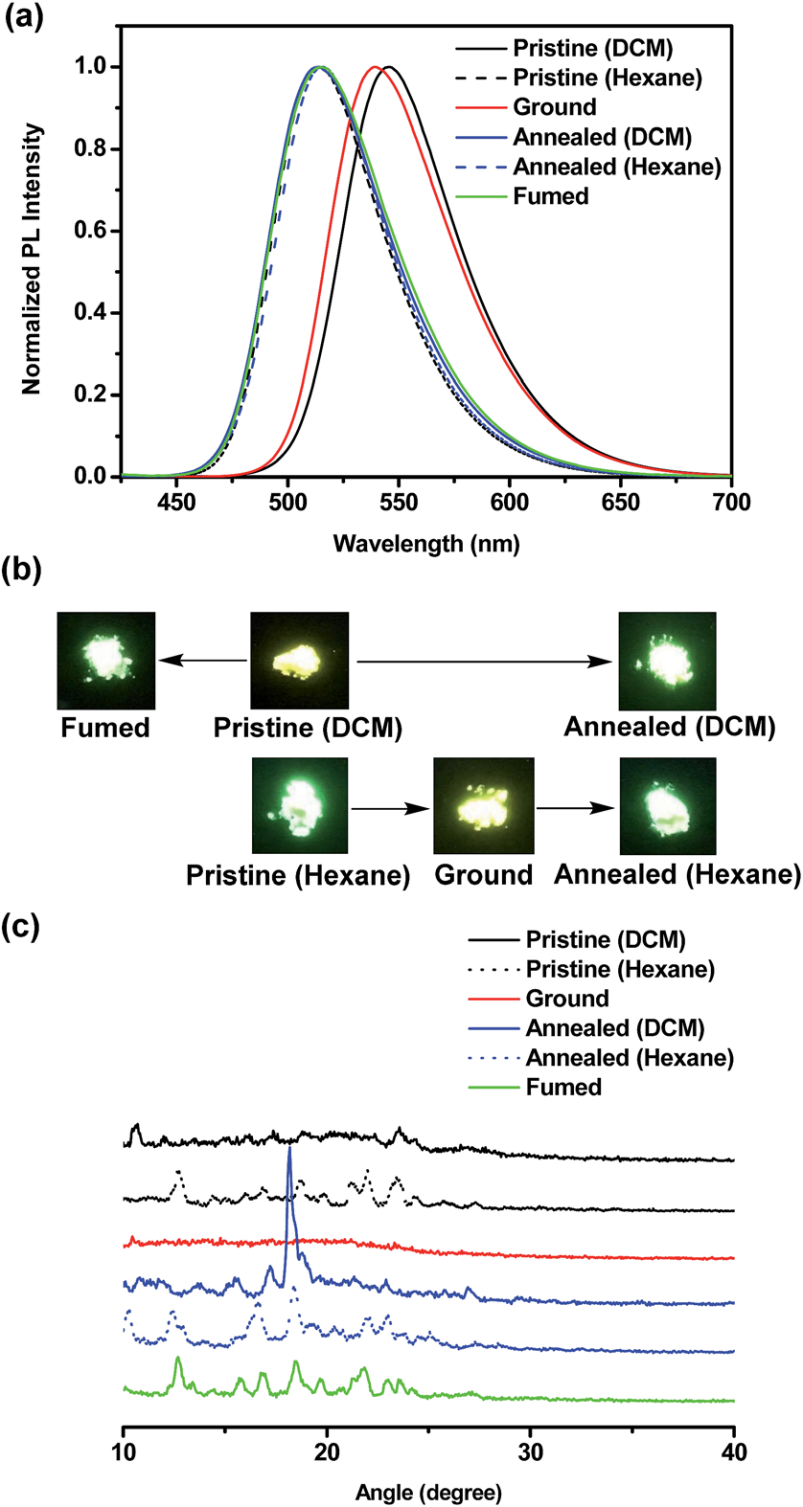
(a) Normalized PL spectra (b) pictures and (c) PXRD diagrams of BT-2TPE in pristine, ground, annealed and fumed powders.

The mechanochromism of three compounds was investigated by emission spectroscopy. The emission spectra of compound BT-2TPE prepared by different processes were shown in Fig. 7a. Pristine powders obtained by evaporating dichloromethane exhibited an emission maximum at 545 nm. After fuming with dichloromethane vapor for 5 min, the emission maximum was at 515 nm. In addition, after annealing the pristine powders for 20 min, the emission maximum was at 513 nm. Pristine powders obtained by evaporating dichloromethane *via* fuming or annealing process both exhibited a notable hypsochromic shift of around 30 nm with color changes from yellow to green (Fig. 7b) due to a decreased D–A interaction. Pristine powders obtained by evaporating hexane had an emission maximum at 514 nm. After the grinding process, the ground compound revealed an emission maximum of 539 nm which was red-shifted by 25 nm with a color change from green to yellow (Fig. 7b) indicating an enhanced D–A interaction. Further annealing of the ground compound resulted in a shifting of the emission maximum to 516 nm with a color change from yellow to green. This suggested that conjugation or D–A interaction of compounds can be generated by simple grinding process which leads to a notable bathochromic shift. Powder X-ray diffraction (PXRD) measurements were employed to elucidate the micro-structures of the pristine powders and powders through grinding and annealing process. PXRD results (Fig. 7c) revealed that fumed and annealed powders exhibited relatively sharp diffraction peaks compared to that of the pristine powders obtained by evaporating DCM indicating the generation of a crystalline form. Upon grinding pristine powders obtained by evaporating hexane, the sharp peaks disappeared suggesting a transition from the crystalline to the amorphous state. The ground powders were then annealed and the sharp diffraction peaks appeared again indicating the regeneration of the crystalline form.

**Fig. 8:**
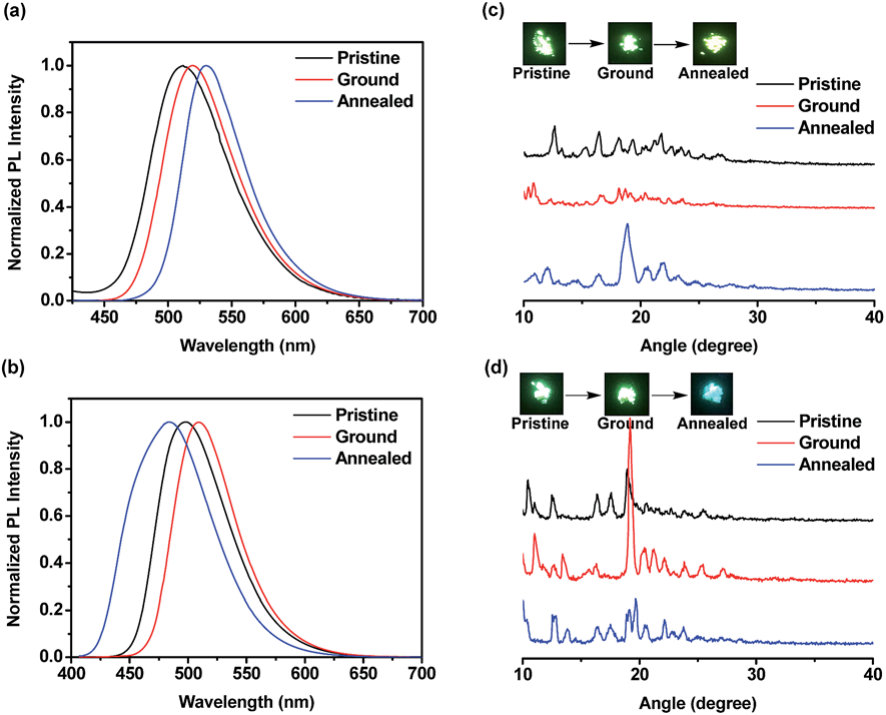
Normalized PL spectra of (a) FBT-2TPE and (b) 2FBT-2TPE and PXRD results and photo images of (c) FBT-2TPE and (d) 2FBT-2TPE in pristine, ground and annealed powders. Photo images of pristine, ground and annealed powders FBT-2TPE and 2FBT-2TPE are shown in the insets.

The emission spectra of compounds FBT-2TPE and 2FBT-2TPE as pristine, ground and annealed powders were shown in Fig. 8a and b. The pristine, ground and annealed powders of FBT-2TPE exhibited an emission maximum at 512, 519 and 530 nm, respectively. In addition, the pristine, ground and annealed powders of 2FBT-2TPE exhibited an emission maximum at 498, 509 and 484 nm, respectively. It should be noted that a pronounced color change from green to blue after annealing of the ground compound 2FBT-2TPE was observed due to a decreased D–A interaction. PXRD results (Fig. 8c) for FBT-2TPE exhibited less intense diffraction peaks after the grinding process and new sharp peaks generated by the annealed process indicating the transition from one crystalline form to amorphous state to another crystalline form. In addition, the ground powders of 2FBT-2TPE (Fig. 8d) showed relatively sharp diffraction peaks compared to pristine powders indicating the high degree of crystallinity. The ground samples were then annealed and a selection of low intensity and new peaks were observed possibly due to the conversion of one crystalline form to another. This suggests that compounds FBT-2TPE and 2FBT-2TPE could form other types of well-organized structures by annealing and grinding processes, respectively compared to that of the pristine powders.^35^

## Experimental

### Materials and general considerations

All chemicals and reagents were purchased from commercial sources (Alfa Aesar, Acros and Sigma-Aldrich) without further purification. Solvents used for spectroscopic measurements were spectrograde. All air sensitive reactions were carried out under an argon or a nitrogen atmosphere. Anhydrous organic solvents were distilled under a nitrogen atmosphere over calcium hydride or sodium/benzophenone. Well-degassed solutions were carried out through bubbling argon for at least 30 min. Column chromatography was performed using Silica Flash G60 70–230 mesh silica. ^1^H, ^1^H–^1^H COSY, ^1^H–^13^C HSQC and ^13^C NMR spectra were recorded on Bruker AVIII HD-600 MHz spectrometer. The chemical shifts were reported in ppm. All coupling constants were reported in hertz (Hz). The following abbreviations were used to indicate multiplicity: s = singlet, d = doublet, dd = doublet of doublets, ddd = doublet of doublet of doublets t = triplet and m = multiplet. Electron impact mass spectra (EI-MS) were recorded on a JEOL JMS-700 spectrometer. Electrospray ionization mass spectra (ESI-MS) were recorded on a Thermo/Finnigan LCQ spectrometer. UV-vis absorption and emission spectra were recorded on a Jasco (V-670) UV-Vis-NIR spectrophotometer and on a Jasco (FP-8500) fluorescence spectrophotometer, respectively. The photoluminescence quantum yields (*Φ*) of compounds were performed in THF and water solutions, respectively, relative to a quinine sulfate (*Φ* = 0.54 in 0.1 N H_2_SO_4_) standard. Thermogravimetric analysis was carried out by using a TA instruments TGA Q-500 under a heating rate of 10 °C min^−1^ and a nitrogen flow rate of 60 mL min^−1^. Differential scanning calorimetry (DSC) was carried out using a PerkinElmer DSC 4000 under a heating and a cooling rate of 10 °C min^−1^ and a nitrogen flow rate of 20 mL min^−1^. Powder X-ray diffraction (PXRD) data was recorded on a D2 PHASER X-ray Diffractometer with Cu Kα radiation (*λ* = 1.5418 Å). Polarized optical microscopy (POM) images were obtained from Olympus BX51 polarized light microscopy.

#### Synthesis of 5-fluorobenzo[*c*][1,2,5]thiadiazole 1

4-Fluoro-*o*-phenylenediamine (2.20 g, 17.4 mmol) was dissolved in a mixture of 200 mL of anhydrous CHCl_3_ and 12 mL of anhydrous Et_3_N under an argon atmosphere and then thionyl chloride (2.8 mL, 36.4 mmol) was added dropwise by syringe. The mixture was refluxed overnight. The reaction mixture was then cooled down to room temperature and most of the CHCl_3_ was evaporated. Deionized water (250 mL) was added to the reaction mixture and then concentrated HCl was added slowly until the pH value was 1.0. Through direct steam distillation was used to collect all condensed liquid, extracted three times then dried over anhydrous MgSO_4_. The solvent was removed by rotary evaporator to give a white solid (0.88 g, 5.7 mmol) in a yield of 33%. ^1^H NMR (600 MHz, CDCl_3_, ppm) *δ*: 7.92 (dd, 1H, *J* = 9.6, 5.4 Hz), 7.56 (dd, 1H, *J* = 8.6, 2.5 Hz), 7.37 (ddd, 1H, *J* = 9.6, 8.6, 2.5 Hz). ^13^C NMR (150 MHz, CDCl_3_, ppm) *δ*: 163.48 (d, ^1^*J*_CF_ = 253 Hz), 154.87 (d, ^3^*J*_CF_ = 14 Hz), 151.95, 122.49 (d, ^3^*J*_CF_ = 11 Hz), 121.27 (d, ^2^*J*_CF_ = 30 Hz), 104.76 (d, ^2^*J*_CF_ = 24 Hz). ^19^F NMR (564 MHz, CDCl_3_, ppm) *δ*: −110.38. EI-MS: calculated for [C_6_H_3_FN_2_S]^+^: *m*/*z* 154, found *m*/*z* 154.

#### Synthesis of 5-fluorobenzo[*c*][1,2,5]thiadiazole 2

A similar procedure was used as that described for the synthesis of compound 1 using 1,2-diamino-4,5-difluoro-benzene (2.52 g, 17.4 mmol) as the starting material. Compound 2 was obtained to give a white solid (0.92 g) in a yield of 31%. ^1^H NMR (600 MHz, CDCl_3_, ppm) *δ*: 7.76 (dd, 2H, *J* = 9.0, 4.5 Hz). ^13^C NMR (150 MHz, CDCl_3_, ppm) *δ*: 153.80 (dd, 2C, ^1^*J*_CF_ = 261 Hz, ^2^*J*_CF_ = 20 Hz), 148.85 (*pseudo* triplet, 2C, ^3^*J*_CF_ = 5 Hz, ^3^*J*_CF_ = 2 Hz), 106.12 (dd, 2C, ^2^*J*_CF_ = 16 Hz, ^3^*J*_CF_ = 5 Hz). ^19^F NMR (564 MHz, CDCl_3_, ppm) *δ*: −128.96. EI-LRMS: calculated for [C_6_H_2_F_2_N_2_S]^+^: *m*/*z* 172, found *m*/*z* 172.

#### Synthesis of 4,7-dibromo-5-fluorobenzo[*c*][1,2,5]thiadiazole 3

Compound 1 (0.88 g, 5.7 mmol) and 12 mL of 48% HBr_(aq)_ were charged into a two-neck flask and then bromine was added dropwise with a dropping funnel. The suspension was heated to 120 °C for 2 days. The suspension was cooled to room temperature and was quenched by saturated aqueous NaHSO_3_. The mixture was extracted with DCM three times and the combined organic layers were dried over anhydrous MgSO_4_. The solvent was removed by rotary evaporator and the residue was purified by column chromatography (petroleum ether : DCM = 8 : 2) to give a white solid (0.82 g) in a yield of 46%. ^1^H NMR (600 MHz, CDCl_3_, ppm) *δ*: 7.79 (d, 1H, *J*_H–F_ = 8.3 Hz). ^13^C NMR (150 MHz, CDCl_3_, ppm) *δ*: 160.40 (d, ^1^*J*_CF_ = 256 Hz), 154.87 (d, ^3^*J*_CF_ = 14 Hz), 150.33, 123.88 (d, ^2^*J*_CF_ = 32 Hz), 114.00 (d, ^3^*J*_CF_ = 11 Hz), 98.21 (d, ^2^*J*_CF_ = 24 Hz). ^19^F NMR (564 MHz, CDCl_3_, ppm) *δ*: −103.25. EI-MS: calculated for [C_6_H_1_Br_2_FN_2_S]^+^: *m*/*z* 312, found *m*/*z* 312.

#### Synthesis of 4,7-dibromo-5,6-difluorobenzo[*c*][1,2,5]thiadiazole 4

A similar procedure was used as that described for the synthesis of compound 3 using 5-fluorobenzo[*c*][1,2,5]thiadiazole (0.92 g, 5.3 mmol) as the starting material. Compound 4 was obtained to give a white solid (0.82 g) in a yield of 47%. ^1^H NMR (600 MHz, CDCl_3_, ppm) *δ*: no peaks. ^13^C NMR (150 MHz, CDCl_3_, ppm) *δ*: 151.84 (dd, 2C, ^1^*J*_CF_ = 262 Hz, ^2^*J*_CF_ = 22 Hz), 148.85 (s, 2C), 99.35 (dd, 2C, ^2^*J*_CF_ = 16 Hz, ^3^*J*_CF_ = 6 Hz). ^19^F NMR (564 MHz, CDCl_3_, ppm) *δ*: −119.53. EI-MS: calculated for [C_6_Br_2_F_2_N_2_S]^+^: *m*/*z* 330, found *m*/*z* 330.

#### Synthesis of 4,4,5,5-tetramethyl-2-(4-(1,2,2-triphenylvinyl)phenyl)-1,3,2-dioxaborolane 5

1-(4-Bromophenyl)-1,2,2-triphenylethylene (3.00 g, 7.3 mmol), bis(pinacolato)diboron (2.24 g, 8.8 mmol), potassium acetate (2.87 g, 29.2 mmol) and Pd(dppf)Cl_2_ (266 mg, 0.36 mmol) were charged into a two-neck flask and then 30 mL of anhydrous 1,4-dioxane was added by syringe. The reaction mixture was refluxed under an argon atmosphere for 12 hours. After cooling to room temperature, the catalyst was removed by filtration. The filtrate was extracted with DCM three times and the combined organic layers were dried over anhydrous MgSO_4_. After removal of the solvent under reduced pressure, the residue was purified by column chromatography (petroleum ether : ether = 9 : 1) to give a white solid (2.54 g) in a yield of 76%. ^1^H NMR (600 MHz, CDCl_3_, ppm) *δ*: 7.54 (d (AA′BB′), 2H, *J* = 8.1 Hz), 7.12–7.06 (m, 9H), 7.06–6.98 (m, 8H), 1.32 (s, 12H). ^13^C NMR (150 MHz, CDCl_3_, ppm) *δ*: 146.80, 143.73, 143.63, 143.55, 141.42, 140.88, 134.13, 131.38, 131.35, 131.34, 130.73, 127.76, 127.69, 126.59, 126.49, 126.48, 83.71, 24.94. ESI-MS: calculated for [C_32_H_31_BO_2_]^+^: *m*/*z* 459, found *m*/*z* 459.

#### Synthesis of 4,7-bis(4-(1,2,2-triphenylvinyl)phenyl) benzo[*c*][1,2,5]thiadiazole BT-2TPE

4,7-Dibromobenzo[*c*][1,2,5]thiadiazole (0.29 g, 1 mmol), compound 5 (0.82 g, 1.79 mmol), Pd(PPh_3_)_4_ (0.11 g, 0.1 mmol) and potassium carbonate (1.10 g, 8.0 mmol) were charged into a two-neck flask under vacuum for 30 minutes. After purging with argon, 80 mL of toluene, 10 mL of ethanol and 10 mL of water was added by syringe. The reaction mixture was refluxed at 120 °C for 12 hours under an argon atmosphere. After cooling to room temperature, organic solvent was removed by rotary evaporator. The residue was extracted with DCM and water three times and the combined organic layers were dried over anhydrous MgSO_4_. After removal of the solvent under reduced pressure, the residue was purified by column chromatography (DCM : petroleum ether = 6 : 4) to give a yellow solid (0.68 g) in a yield of 85%. ^1^H NMR (600 MHz, CDCl_3_, ppm) *δ*: 7.78 (d(AA′BB′), 4H, *J* = 8.4 Hz), 7.74 (s, 2H), 7.20 (d(AA′BB′), 4H*, J* = 8.4 Hz), 7.18–7.05 (m, 30H). ^13^C NMR (150 MHz, CDCl_3_, ppm) *δ*: 154.01, 143.78, 143.74, 143.65, 143.64, 141.48, 140.51, 136.26, 132.59, 131.59, 131.47, 131.37, 131.35, 128.35, 127.88, 127.81, 127.72, 127.63, 126.58, 126.50, 126.46. HR-MS (EI, [M]^+^): calculated for [C_58_H_40_N_2_S]^+^: *m*/*z* 796.2972, found *m*/*z* 796.2970.

#### Synthesis of 5-fluoro-4,7-bis(4-(1,2,2-triphenylvinyl)phenyl)benzo[*c*][1,2,5]thiadiazole FBT-2TPE

A similar procedure was used as that described for the synthesis of BT-2TPE using compound 3 (0.25 g, 0.8 mmol) as the starting material. The compound FBT-2TPE was obtained to give a yellow solid (0.42 g) in a yield of 64%. ^1^H NMR (600 MHz, DCM-d_2_, ppm) *δ*: 7.79 (d(AA′BB′), 2H, *J* = 8.4 Hz), 7.62 (d, 1H, *J*_H–F_ = 11.8 Hz), 7.60 (d(AA′BB′), 2H, *J* = 8.4 Hz), 7.23–7.04 (m, 34H). ^13^C NMR (150 MHz, CDCl_3_, ppm) *δ*: 133.97, 133.34, 133.27, 131.71, 131.48, 131.43, 131.36, 131.34, 131.31, 131.20, 129.83, 129.81, 129.22, 128.38, 127.84, 127.79, 127.76, 127.72, 127.65, 127.62, 126.67, 126.61, 126.57, 126.54, 126.50, 126.46, 119.51, 119.30, 117.11, 117.01. ^19^F NMR (564 MHz, CDCl_3_, ppm) *δ*: −115.14. HR-MS (EI, [M]^+^): calculated for [C_58_H_39_FN_2_S]^+^: *m*/*z* 814.2818, found *m*/*z* 814.2817.

#### Synthesis of 5,6-difluoro-4,7-bis(4-(1,2,2-triphenylvinyl)phenyl)benzo[*c*][1,2,5]thiadiazole 2FBT-2TPE

A similar procedure was used as that described for the synthesis of BT-2TPE using compound 4 (0.25 g, 0.75 mmol) as the starting material. The compound 2FBT-2TPE was obtained to give a yellow solid (0.25 g) in a yield of 38%. ^1^H NMR (600 MHz, CDCl_3_, ppm) *δ*: 7.61 (d(AA′BB′), 4H, *J* = 8.4 Hz), 7.22 (d(AA′BB′), 4H, *J* = 8.4 Hz), 7.20–7.05 (m, 30H). ^13^C NMR (150 MHz, CDCl_3_, ppm) *δ*: 151.27, 151.13, 150.39, 150.37, 150.35, 149.55, 149.42, 144.48, 143.60, 143.46, 143.45, 141.89, 140.33, 131.45, 131.35, 131.34, 131.32, 129.79, 128.25, 127.81, 127.76, 127.64, 126.68, 126.56, 126.53, 118.33, 118.31, 118.26, 118.24. ^19^F NMR (564 MHz, CDCl_3_, ppm) *δ*: −113.79. HR-MS (EI, [M]^+^): calculated for [C_58_H_38_F_2_N_2_S]^+^: *m*/*z* 832.2724, found *m*/*z* 832.2725.

## Conclusions

Fluorinated BT linked with TPE were successfully synthesized by a Suzuki–Miyaura cross-coupling reaction using Pd(PPh_3_)_4_ as the catalyst and K_2_CO_3_ as a base to give the compounds in a moderate yield. All compounds exhibited aggregation-induced emission characteristics upon increasing the water fraction over 60% in the THF/water mixtures. In addition, the emission maximum was blue-shifted when the water content reached 90% compared to the THF solution. The effect of fluorine atoms attached to the central BT unit makes these D–A–D molecules force to be planar which led to a more significant blue-shifted in absorption and emission maximum in the aggregate state. Interestingly, the emission maximum intensity of the compound containing difluorinated BT bonded to two TPE in 90% water content was found to be 2.5 times higher than that in THF solution which reveal a significant increase in fluorescence compared to that of the unsubstituted and monofluorinated substituted BT. Surprisingly, single crystal with ladder-like packing structures as well as liquid crystal mesophases were observed only for compound comprising of difluorinated BT and TPE. This molecule with the unique feature can be used as a key component in the liquid crystal devices, organic light-emitting diodes and organic field-effect transistors. All compounds showed remarkable solvatochromic effects in various selected solvents with different polarities and revealed notably emission color changes. The powder XRD results and the mechanochromism of the compounds suggested that the solid state structures change from the one form to another. Theses compounds will be of tremendous interest to those chemists and physicists interested in fluorescence materials and sensors in general. In addition, fluorescence and sensing ability of compounds including unsubstituted and fluorinated substituted BT bonded to two TPE for explosives analytes is currently under investigation.

## Conflicts of interest

There are no conflicts to declare.

## Supplementary Material

RA-008-C8RA01448E-s001

RA-008-C8RA01448E-s002

## References

[cit1] Kim H. N., Guo Z., Zhu W., Yoon J., Tian H. (2011). Chem. Soc. Rev..

[cit2] Pu L. (2004). Chem. Rev..

[cit3] Wang J., Qian X. (2006). Org. Lett..

[cit4] Acharyya K., Mukherjee P. S. (2014). Chem. Commun..

[cit5] Ding Y., Tang Y., Zhua W., Xie Y. (2015). Chem. Soc. Rev..

[cit6] Thomas III S. W., Joly G. D., Swager T. M. (2007). Chem. Rev..

[cit7] Chen C.-T. (2004). Chem. Mater..

[cit8] Qian G., Zhong Z., Luo M., Yu D., Zhang Z., Wang Z. Y., Ma D. (2009). Adv. Mater..

[cit9] Yoshii R., Hirose A., Tanaka K., Chujo Y. (2014). Chem.–Eur. J..

[cit10] Gao Y., Feng G., Jiang T., Goh C., Ng L., Liu B., Li B., Yang L., Hua J., Tian H. (2015). Adv. Funct. Mater..

[cit11] Yoshii R., Tanaka K., Chujo Y. (2014). Macromolecules.

[cit12] Liu M., Gao P., Wan Q., Deng F., Wei Y., Zhang X. (2017). Macromol. Rapid Commun..

[cit13] Xiong J.-B., Feng H.-T., Sun J.-P., Xie W.-Z., Yang D., Liu M., Zheng Y.-S. (2016). J. Am. Chem. Soc..

[cit14] Salimimarand M., La D. D., Kobaisi M. A., Bhosale S. V. (2017). Sci. Rep..

[cit15] Hong Y., Xiong H., Lam J. W. Y., Häußler M., Liu J., Yu Y., Zhong Y., Sung H. H. Y., Williams I. D., Wong K. S., Tang B. Z. (2010). Chem.–Eur. J..

[cit16] Ekbote A., Han S. H., Jadhav T., Mobin S. M., Lee J. Y., Misra R. (2018). J. Mater. Chem. C.

[cit17] Ekbote A., Jadhav T., Misra R. (2017). New J. Chem..

[cit18] Misra R., Jadhav T., Dhokale B., Mobin S. M. (2014). Chem. Commun..

[cit19] Li C., Luo X., Zhao W., Li C., Liu Z., Bo Z., Dong Y., Dong Y. Q., Tang B. Z. (2013). New J. Chem..

[cit20] Li M., Yao W., Chen J., Lu H., Zhao Y., Chen C. (2014). J. Mater. Chem. C.

[cit21] Neto B. A. D., Lapis A. A. M., da Silva Júnior E. N., Dupont J. (2013). Eur. J. Org. Chem..

[cit22] Wang Y., Xin X., Lu Y., Xiao T., Xu X., Zhao N., Hu X., Ong B. S., Ng S. C. (2013). Macromolecules.

[cit23] Wang L., Yin L., Ji C., Li Y. (2015). Dyes Pigm..

[cit24] Zhao Z., Deng C., Chen S., Lam J. W. Y., Qin W., Lu P., Wang Z., Kwok Z., Ma Y., Qiu H., Tang B. Z. (2011). Chem. Commun..

[cit25] Dou C., Chen D., Iqbal J., Yuan Y., Zhang H., Wang Y. (2011). Langmuir.

[cit26] Jadhav T., Dhokale B., Misra R. (2015). J. Mater. Chem. C.

[cit27] Cho N., Song K., Lee J. K., Ko J. (2012). Chem.–Eur. J..

[cit28] Buncel E., Rajagopal S. (1990). Acc. Chem. Res..

[cit29] Reichard C. (1994). Chem. Rev..

[cit30] Zhang X., Ma Z., Yang Y., Zhang X., Jia X., Wei Y. (2014). J. Mater. Chem. C.

[cit31] Jadhav T., Dhokale B., Patil Y., Mobin S. M., Misra R. (2016). J. Phys. Chem. C.

[cit32] Irie M. (2000). Chem. Rev..

[cit33] Kishimura A., Yamashita T., Yamaguchi K., Aida T. (2005). Nat. Mater..

[cit34] Kinami M., Crenshaw B. R., Weder C. (2006). Chem. Mater..

[cit35] Sagara Y., Mutai T., Yoshikawa I., Araki K. (2007). J. Am. Chem. Soc..

[cit36] Ooyama Y., Ito G., Fukuoka H., Nagano T., Kagawa Y., Imae I., Komaguchi K., Harima Y. (2010). Tetrahedron.

[cit37] Ooyama Y., Harima Y. (2011). J. Mater. Chem..

[cit38] Sagara Y., Kato T. (2008). Angew. Chem., Int. Ed..

[cit39] Yuan M., Wang D., Xue P., Wang W., Wang J., Tu Q., Liu Z., Liu Y., Zhang Y., Wang J. (2014). Chem. Mater..

[cit40] Yu H., Ren W., Lu H., Liang Y., Wang Q. (2016). Chem. Commun..

[cit41] He Z., Zhang L., Mei J., Zhang T., Lam J. W. Y., Shuai Z., Dong Y. Q., Tang B. Z. (2015). Chem. Mater..

[cit42] Li H., Zhang X., Chi Z., Xu B., Zhou W., Liu S., Zhang Y., Xu J. (2011). Org. Lett..

[cit43] Zhou X., Li H., Chi Z., Zhang X., Zhang J., Xu B., Zhang Y., Liu S., Xu J. (2012). New J. Chem..

[cit44] Jadhav T., Dhokale B., Patil Y., Mobin S. M., Misra R. (2016). J. Phys. Chem. C.

[cit45] Kim S.-K., Park Y.-I., Kang I.-N., Park J.-W. (2007). J. Mater. Chem..

[cit46] Chen S., Li Y., Yang W., Chen N., Liu H., Li Y. (2010). J. Phys. Chem. C.

[cit47] Hu R., Lager E., Aguilar-Aguila A., Liu J., Lam J. W. Y., Sung H. H. Y., Williams I. D., Zhong Y., Wong K. S., Pena-Cabrera E., Tang B. Z. (2009). J. Phys. Chem. C.

