# Supplementary material for: Synthesis, characterization, aggregation-induced emission, solvatochromism and mechanochromism of fluorinated benzothiadiazole bonded to tetraphenylethenes
Source: RSC Adv. 2018 Apr 3;8(23):12619–27. doi: 10.1039/c8ra01448e (PMC9079628; doi:10.1039/c8ra01448e)
Supplement: RA-008-C8RA01448E-s001 [file RA-008-C8RA01448E-s001.pdf]

Electronic Supplementary Material (ESI) for RSC Advances.
This journal is © The Royal Society of Chemistry 2018

Synthesis, Characterization, Aggregation-induced Emission, Solvatochromism and Mechanochromism of Fluorinated Benzothiadiazole Bonded to Tetraphenylathenes

Chin-Yang Yu*, Chia-Chieh Hsu, Hsi-Chen Weng

Department of Materials Science and Engineering, National Taiwan University of Science and Technology, 43, Section 4, Keelung Road, Taipei, 10607, Taiwan

Tel: +886 2 27376525

Fax: +886 2 27376544

E-mail: cyyu@mail.ntust.edu.tw

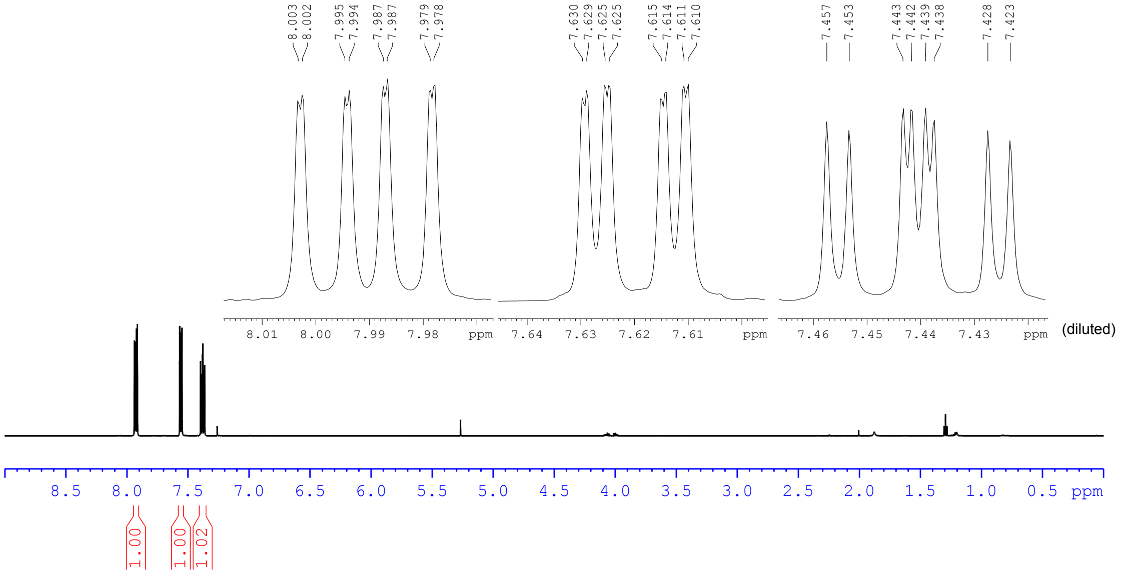

Figure S1. ^{1}H NMR spectrum of compound **1**.

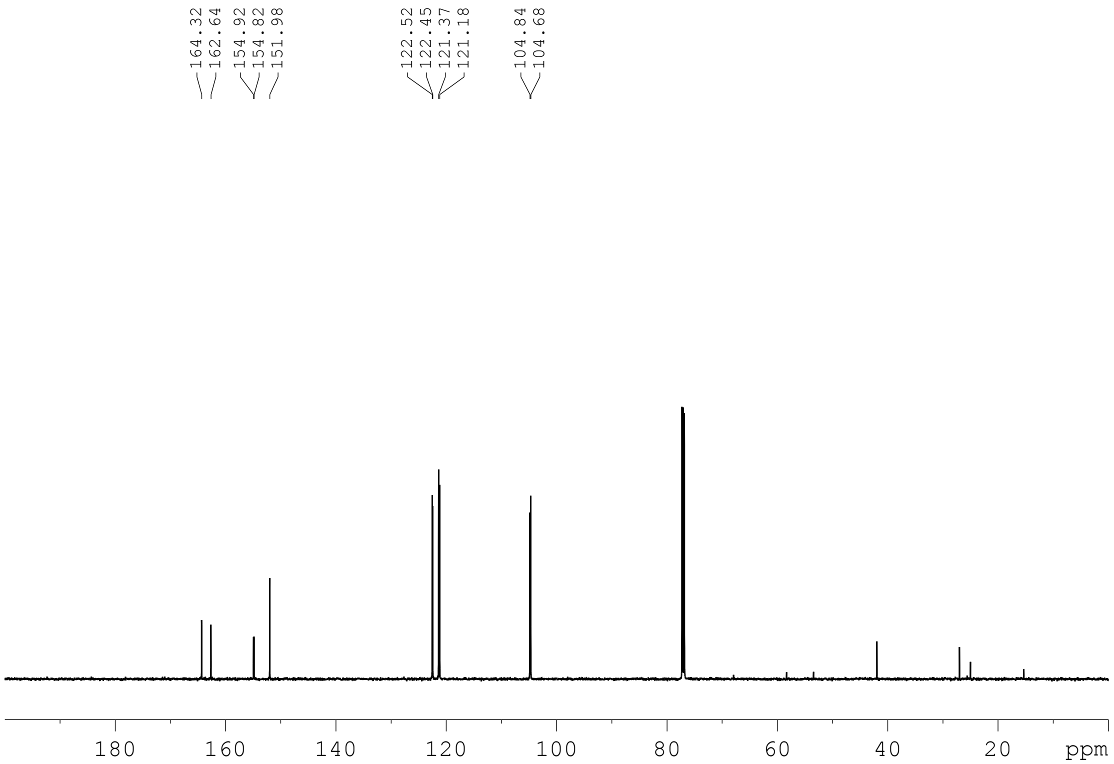

Figure S2. ^{13}C NMR spectrum of compound **1**.

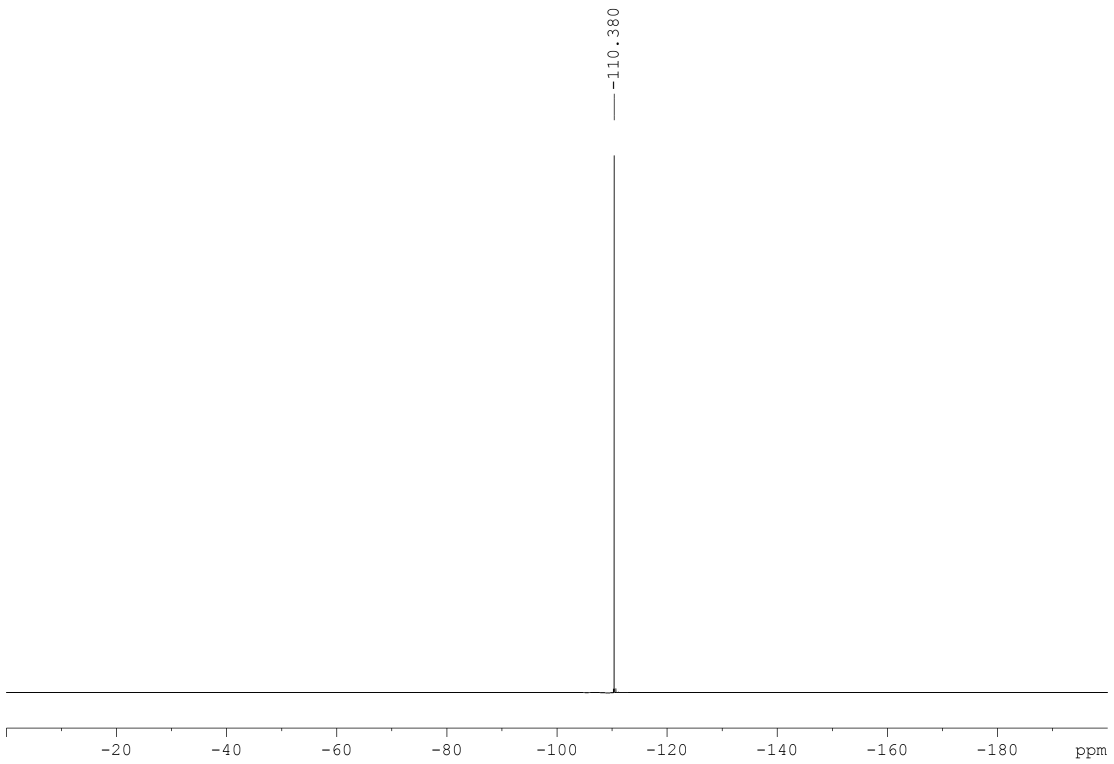

Figure S3. ^{19}F NMR spectrum of compound **1**.

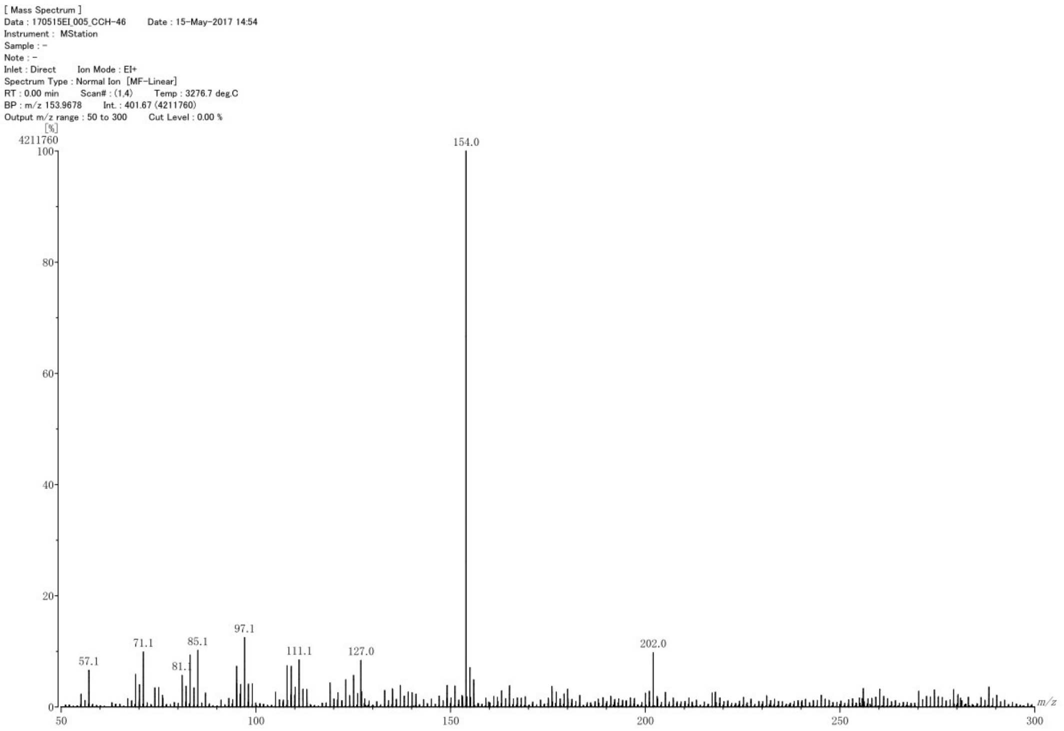

Figure S4. EI Mass spectrum of compound **1**.

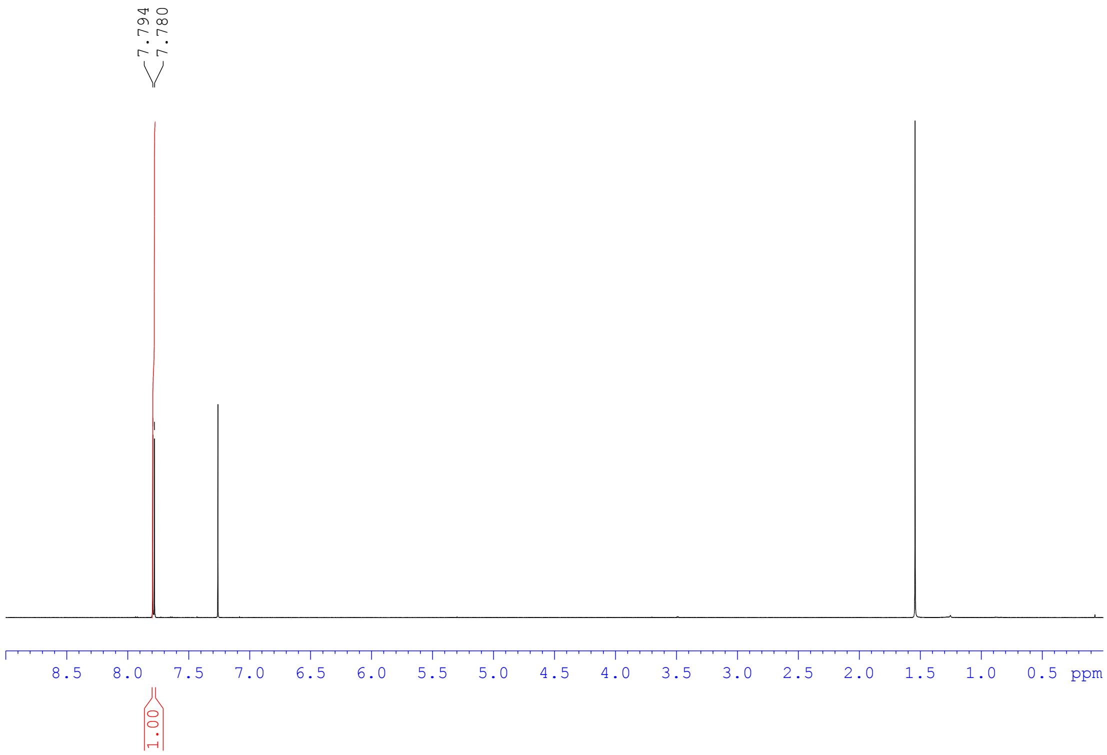

Figure S5. ^{1}H NMR spectrum of compound **2**.

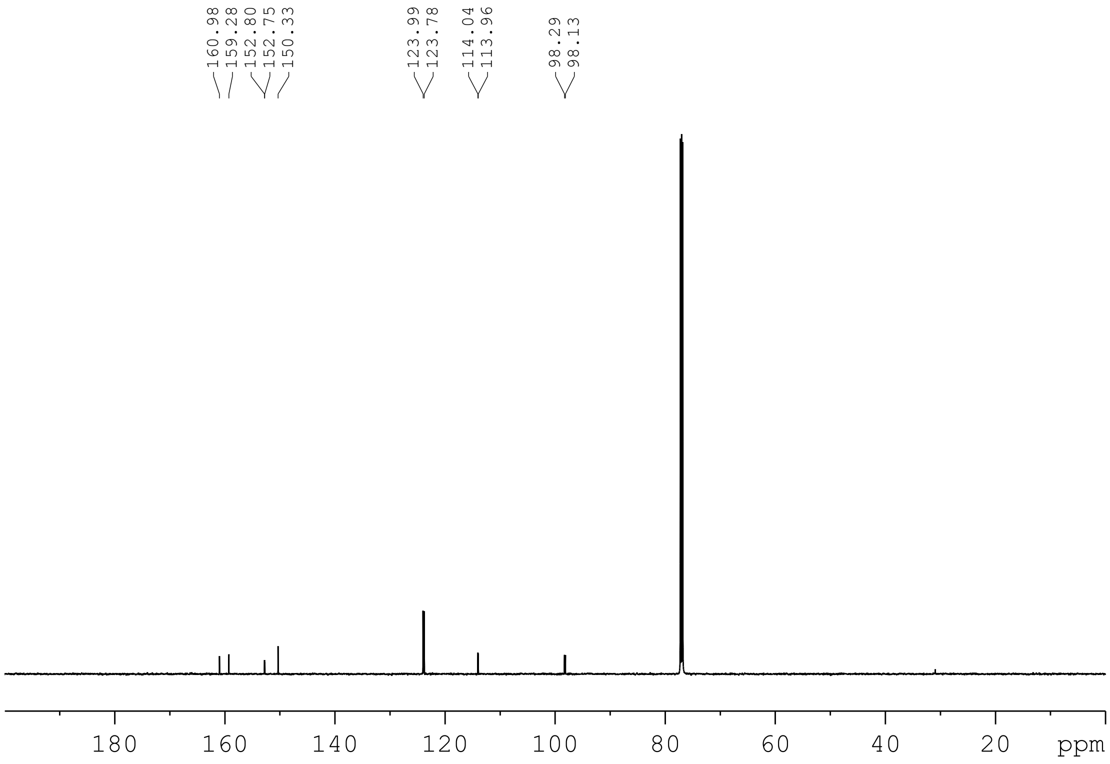

Figure S6. ^{13}C NMR spectrum of compound **2**.

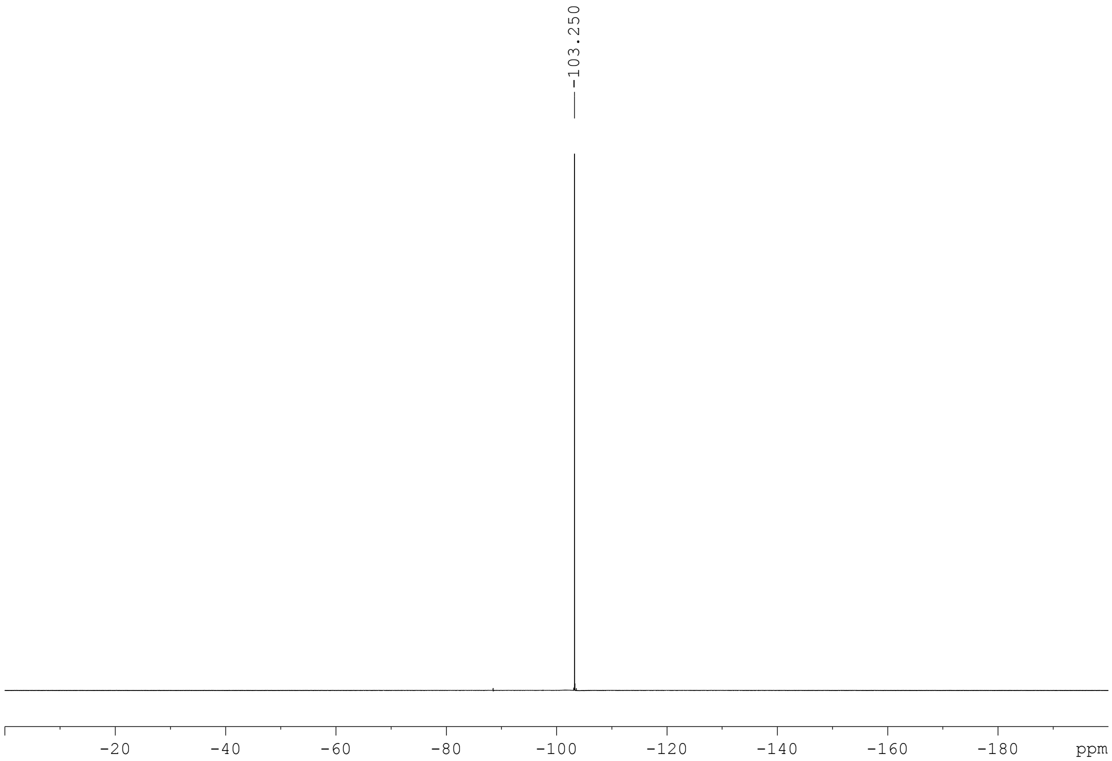

Figure S7. ^{19}F NMR spectrum of compound **2**.

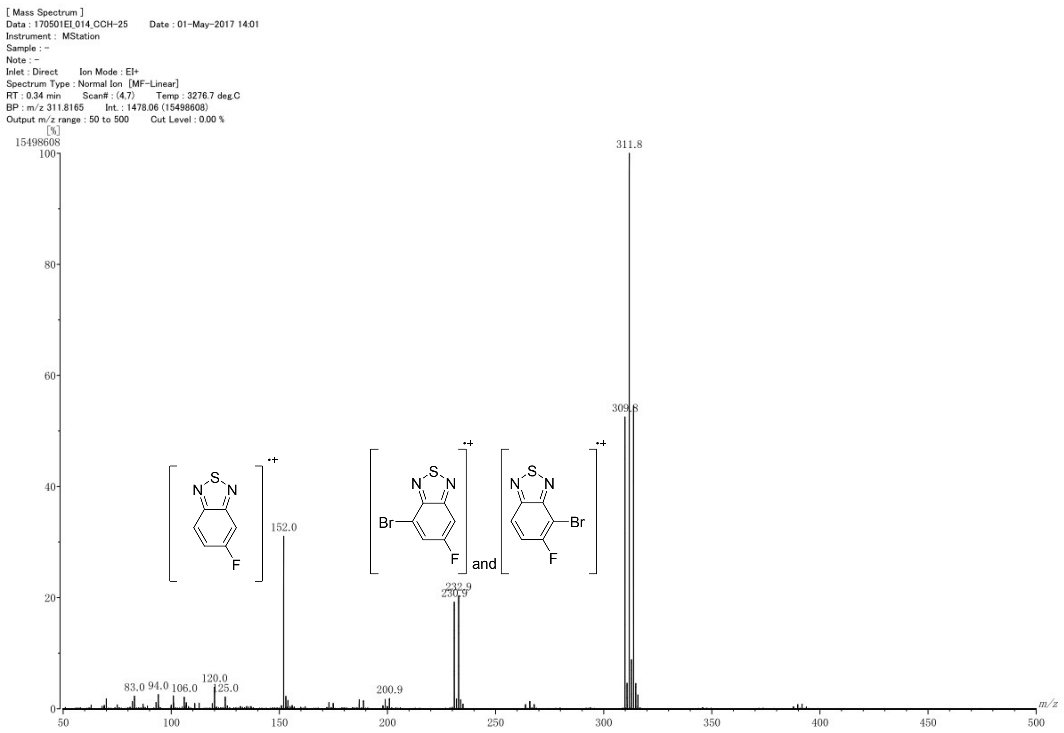

Figure S8EI Mass spectrum of compound **2**.

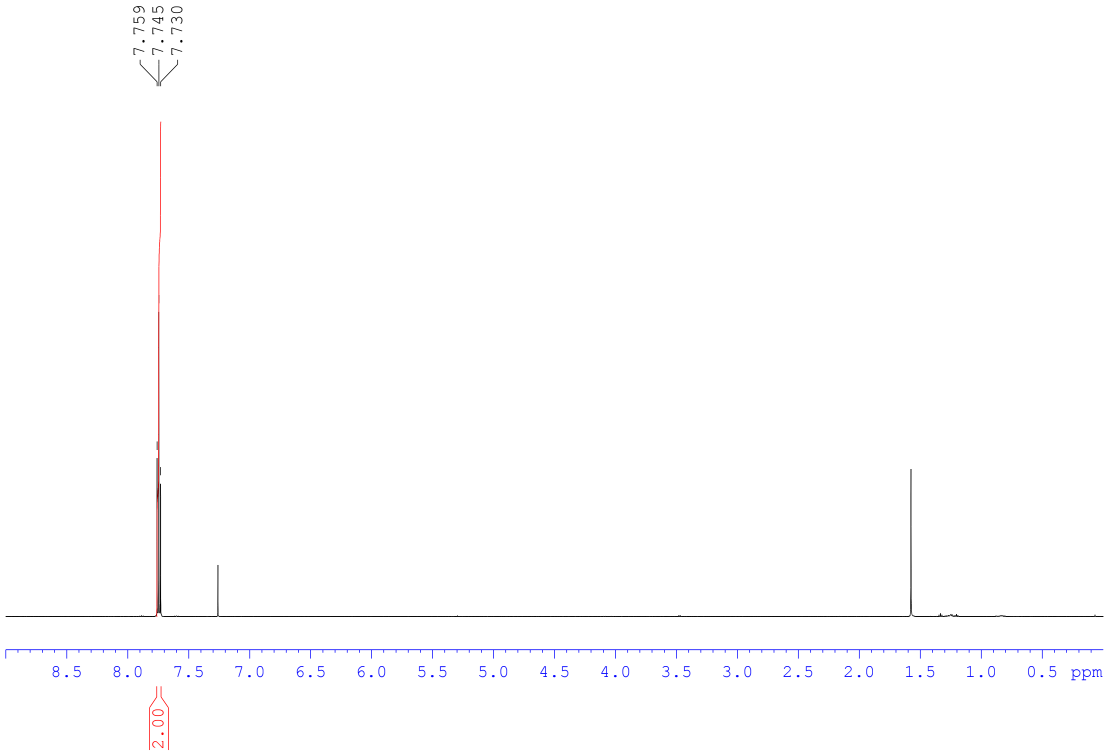

Figure S9. ^{1}H NMR spectrum of compound **3**.

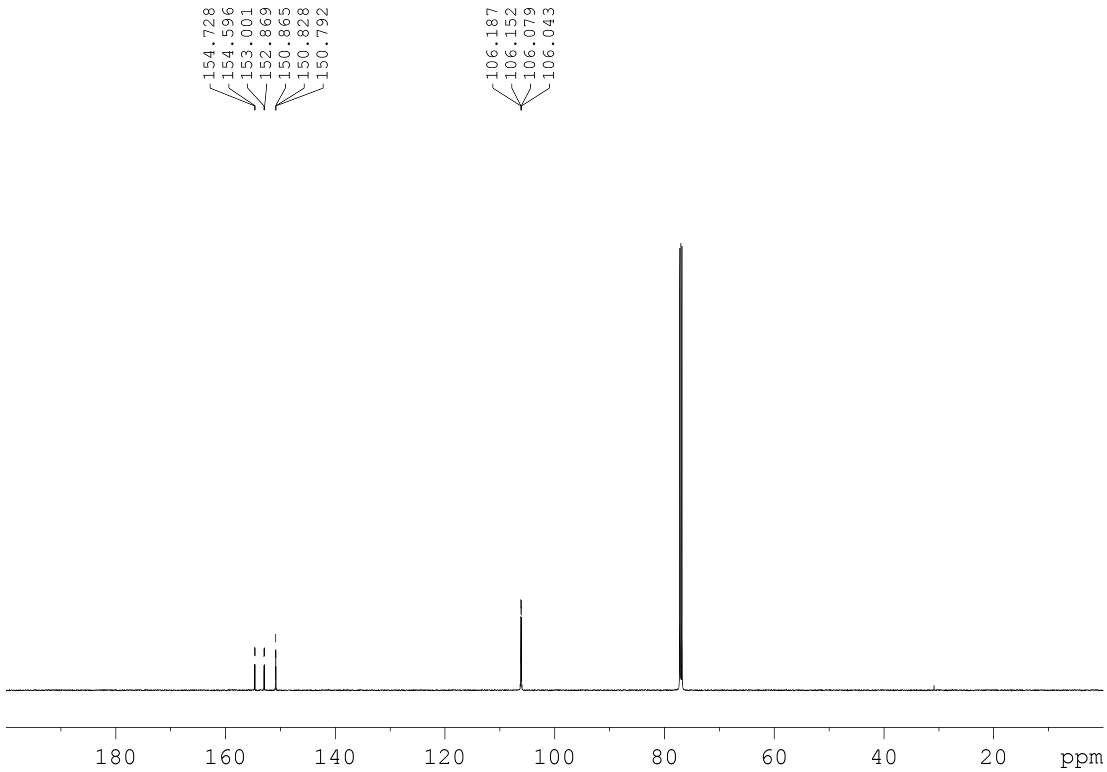

Figure S10. ^{13}C NMR spectrum of compound **3**.

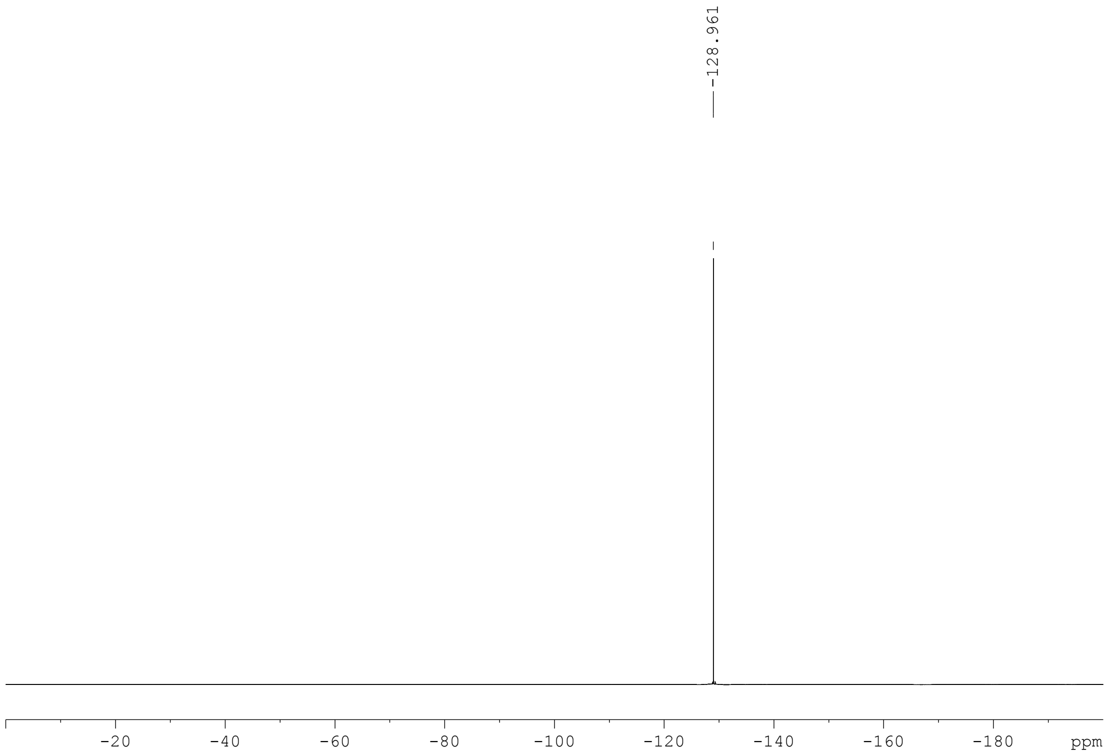

Figure S11. ^{19}F NMR spectrum of compound **3**.

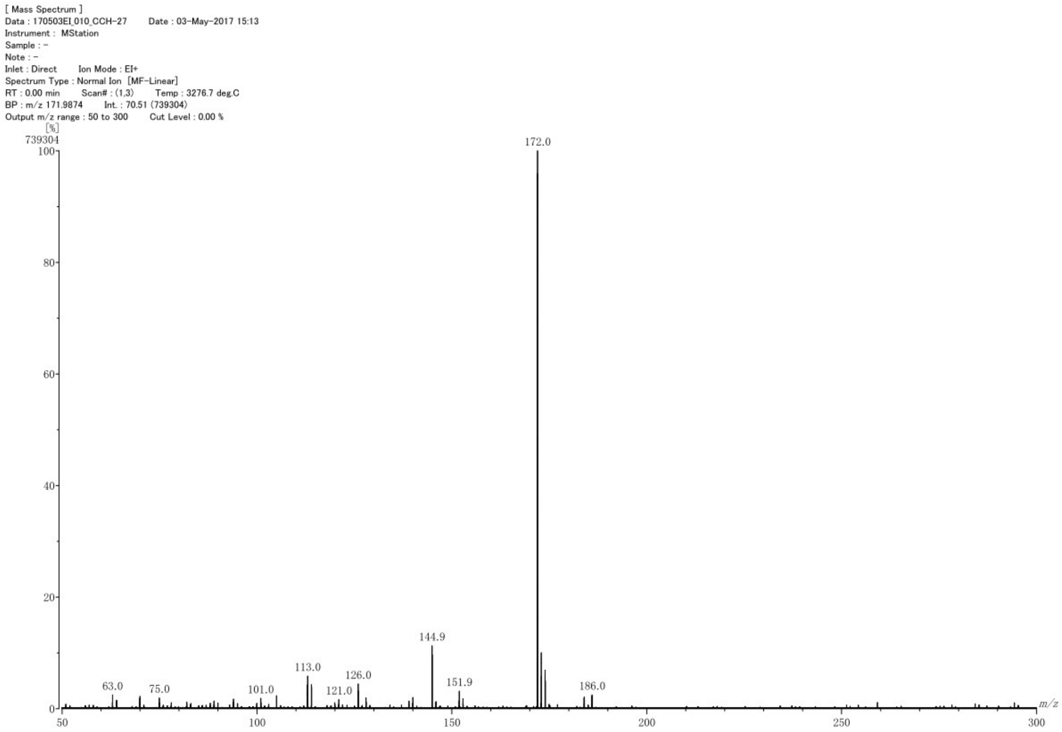

Figure S12. LREI Mass spectrum of compound **3**.

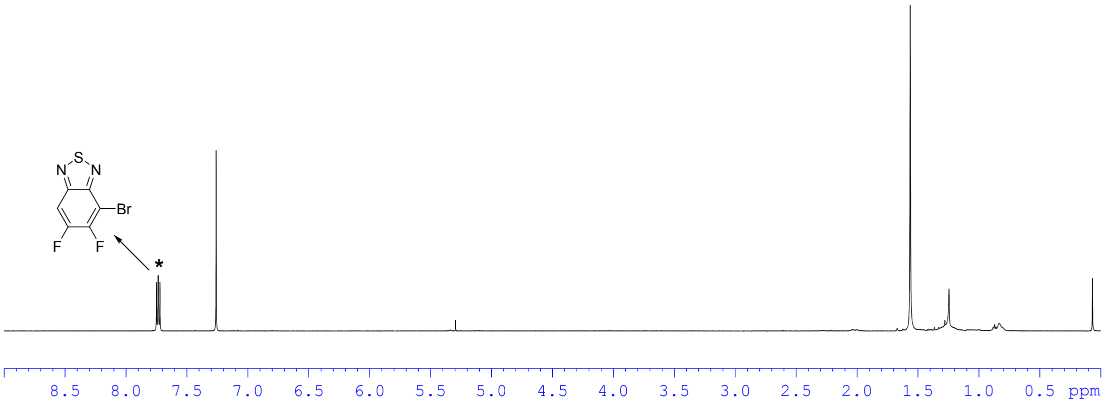

Figure S13. ^{1}H NMR spectrum of compound **4**. (*estimated impurity in high the concentration)

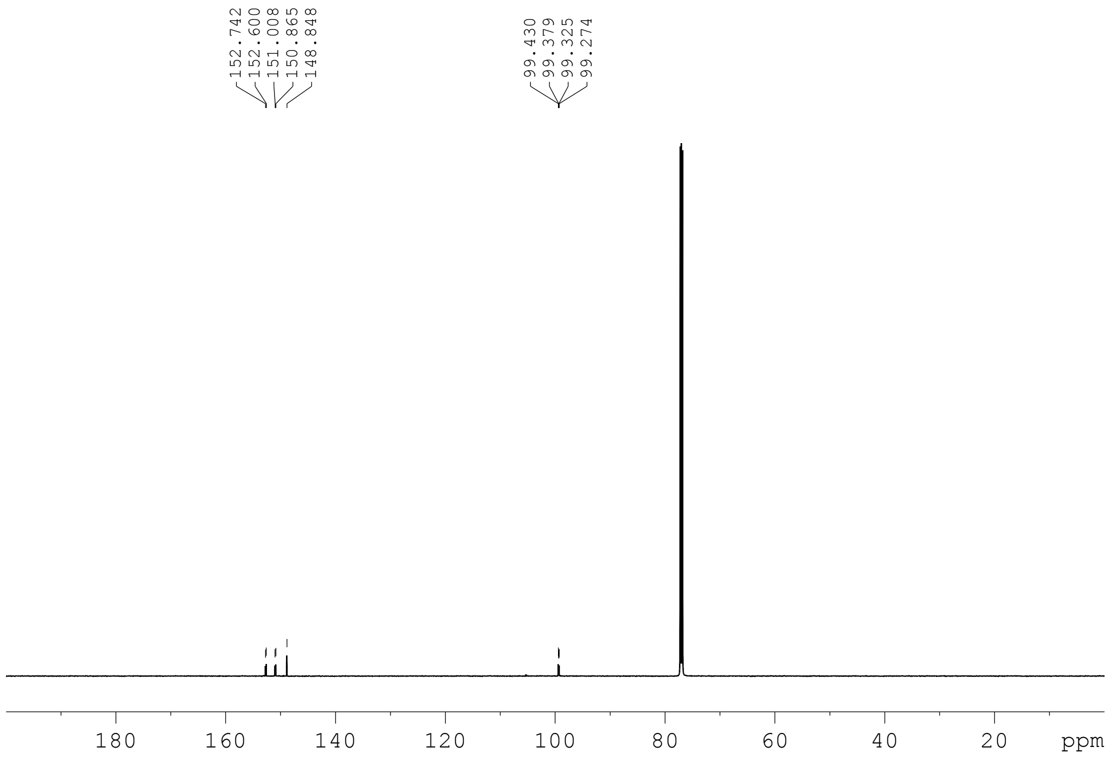

Figure S14. ^{13}C NMR spectrum of compound **4**.

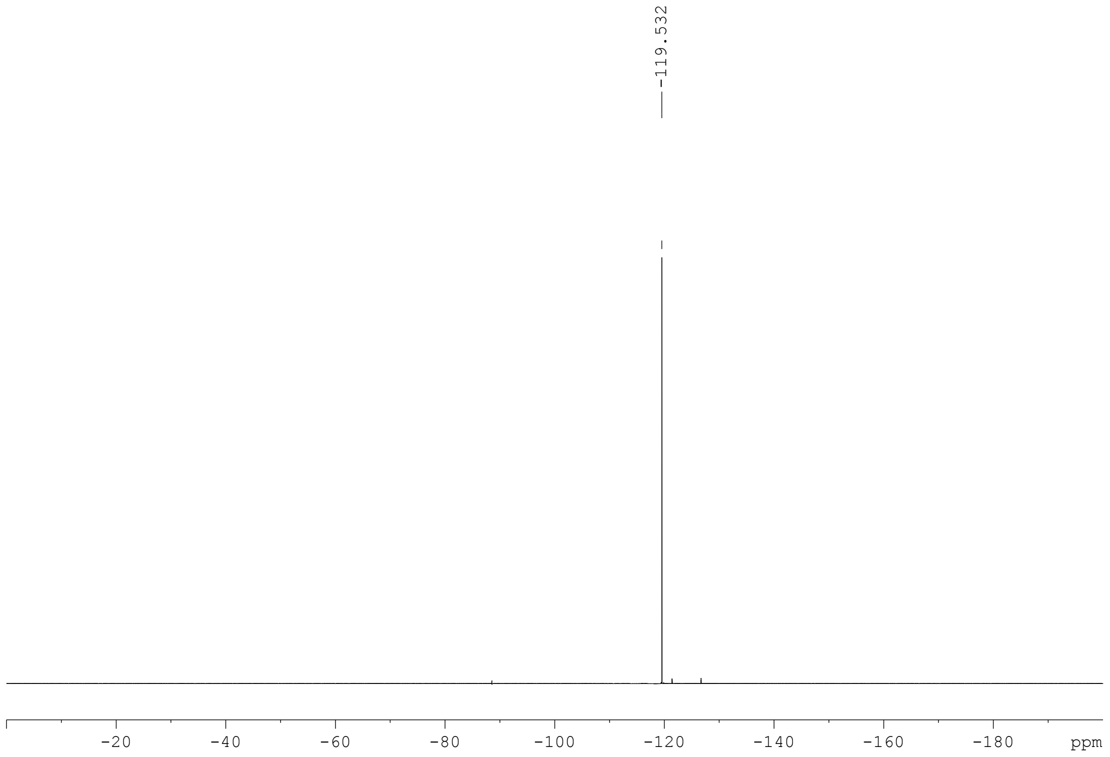

Figure S15. ^{19}F NMR spectrum of compound **4**.

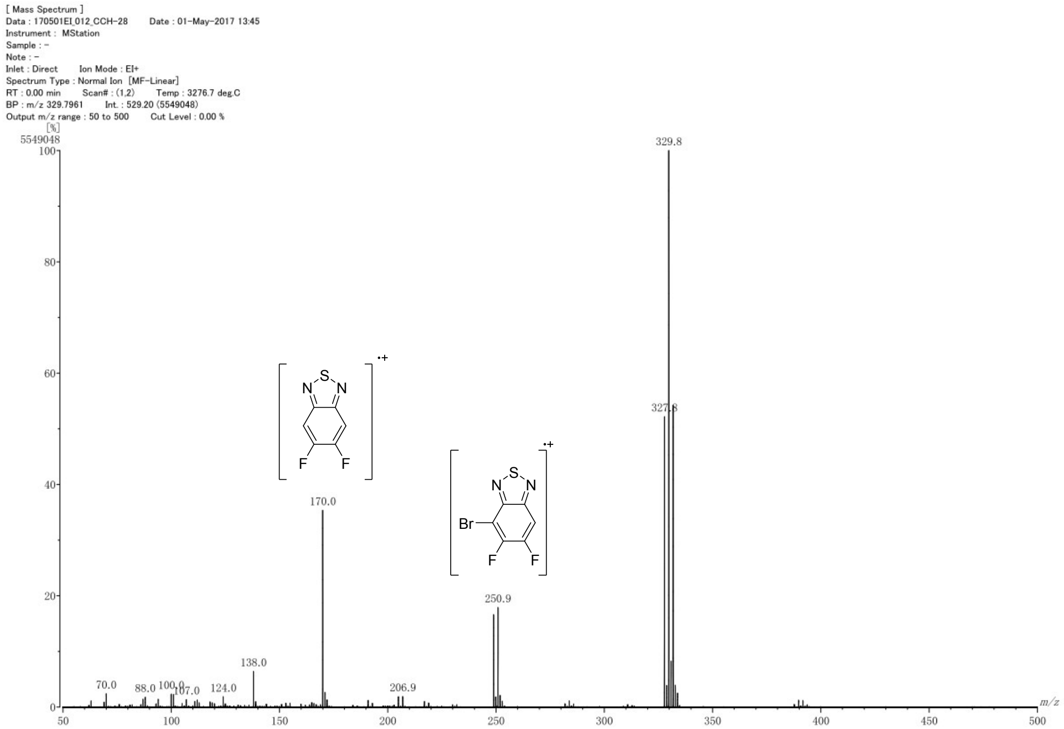

Figure S16. LREI Mass spectrum of compound **4**.

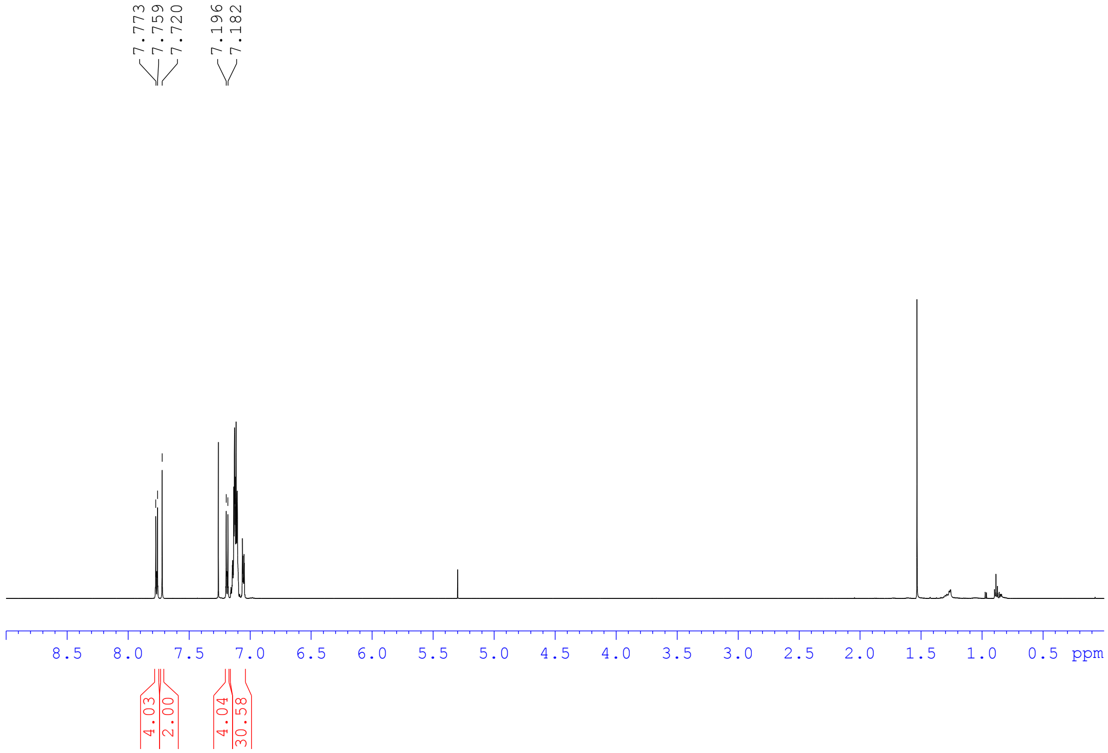

Figure S17. ^{1}H NMR spectrum of compound **BT-2TPE**.

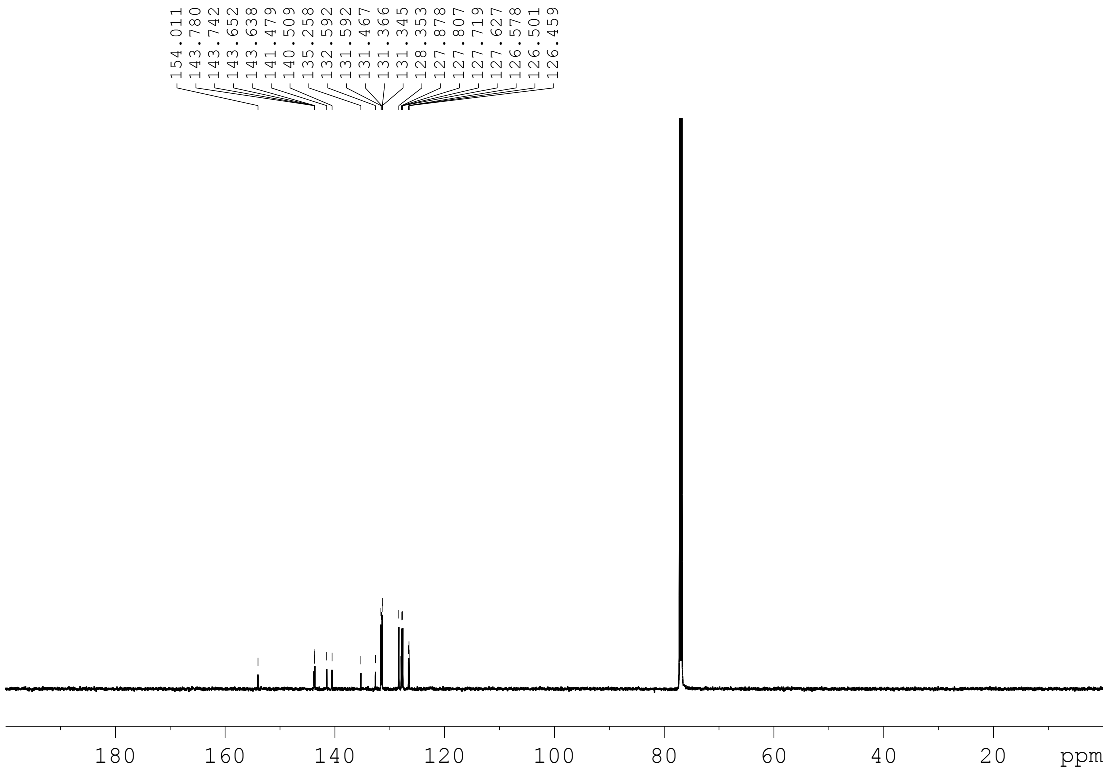

Figure S18. ^{13}C NMR spectrum of compound **BT-2TPE**.

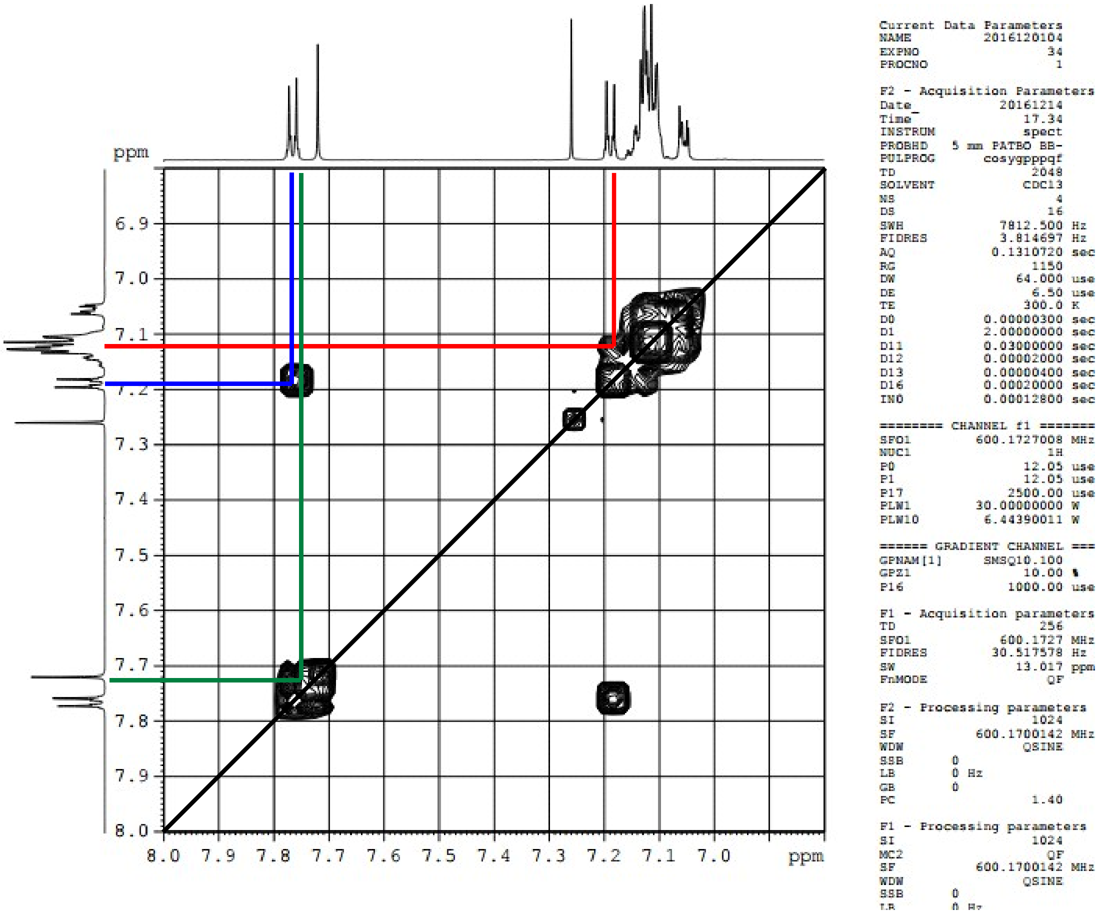

Figure S19. ^1H-^1H COSY NMR spectrum of **BT-2TPE**. (aromatic region)

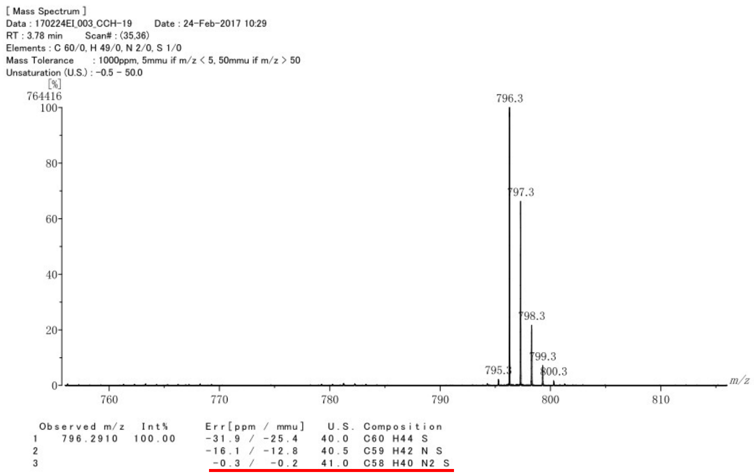

Figure S20. HR-EI Mass spectrum of compound **BT-2TPE**.

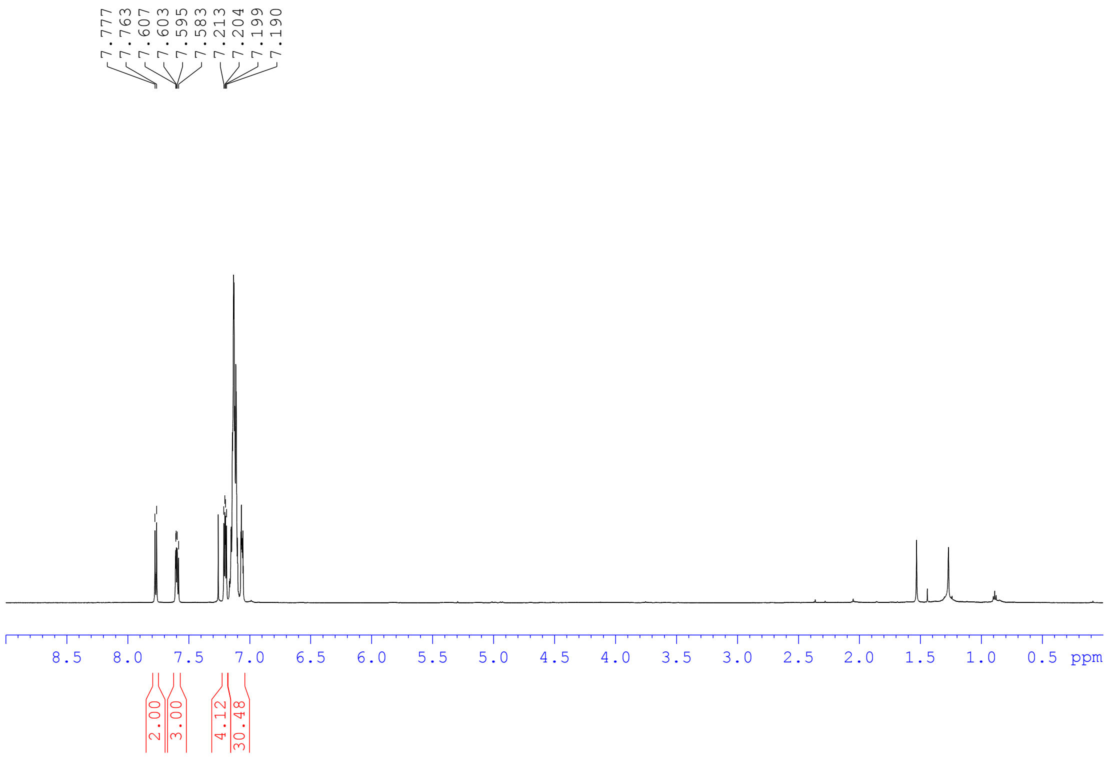

Figure S21. ^{1}H NMR spectrum of compound **FBT-2TPE**.

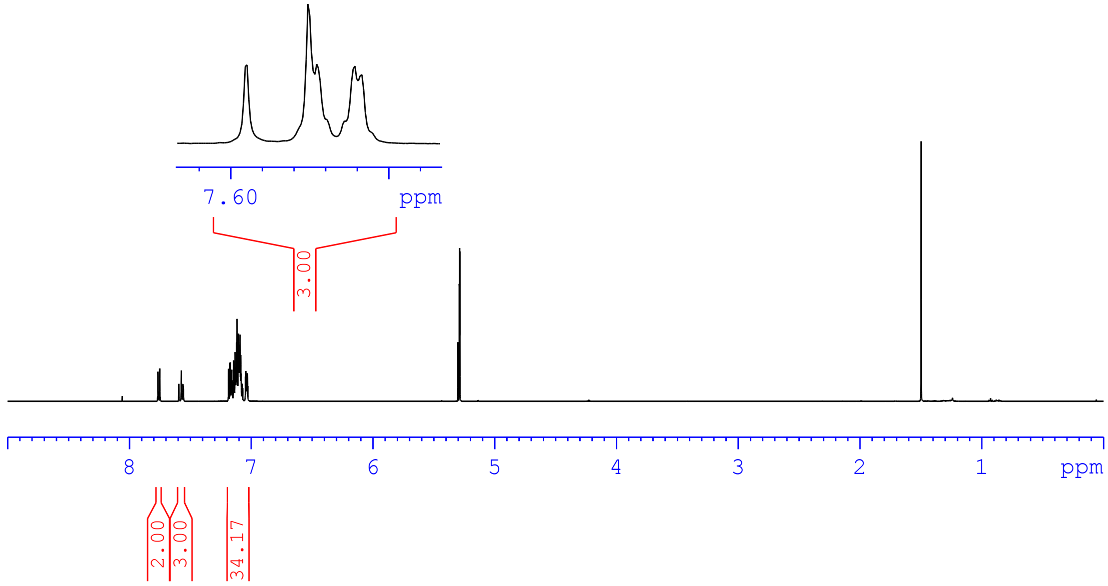

Figure S22. ^{1}H NMR spectrum of compound **FBT-2TPE** (DCM-d_2).

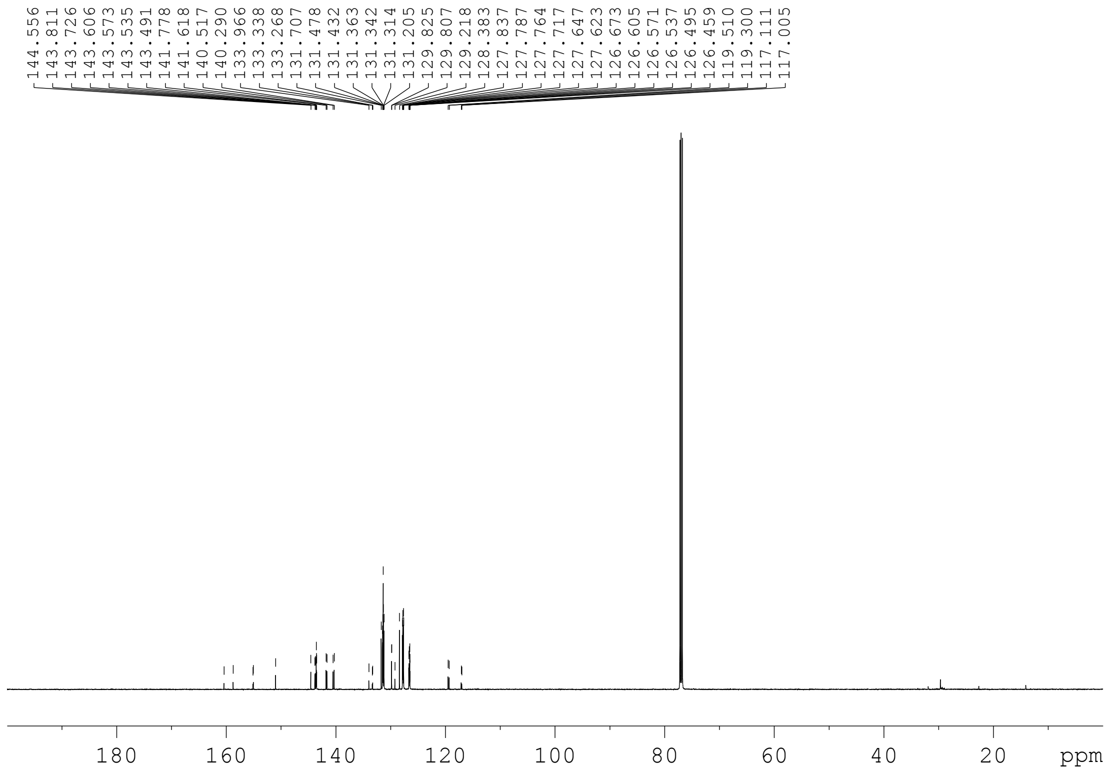

Figure S23. ^{13}C NMR spectrum of compound **FBT-2TPE**.

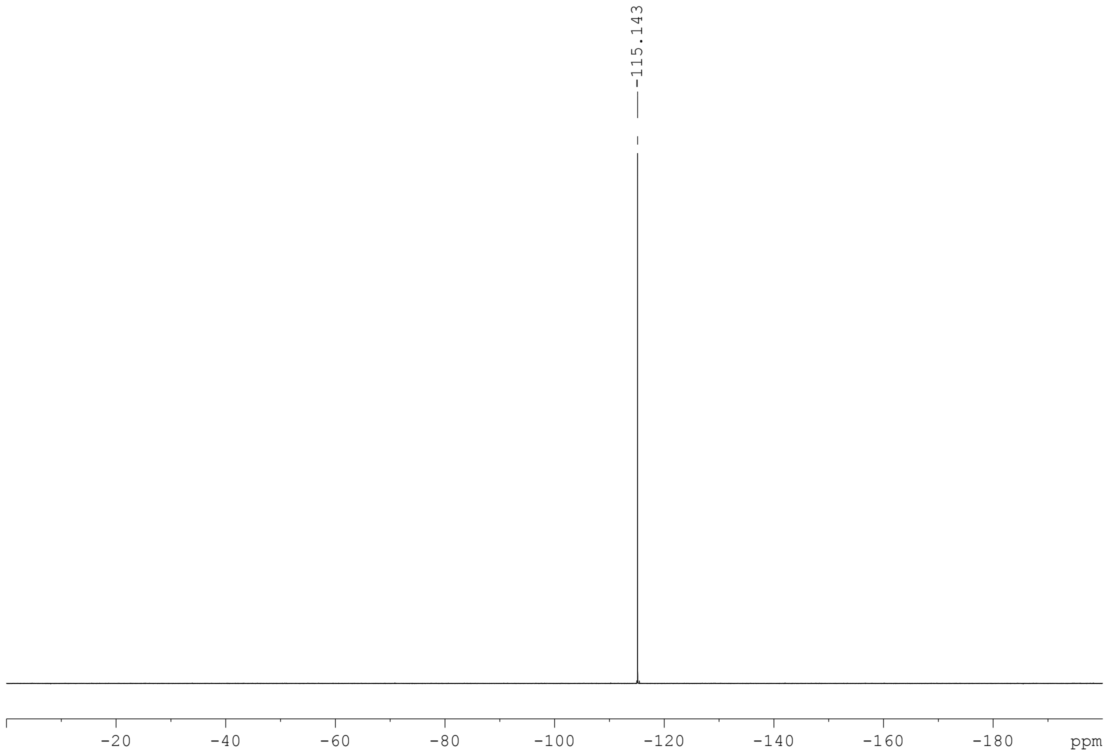

Figure S24. ^{19}F NMR spectrum of compound **FBT-2TPE**.

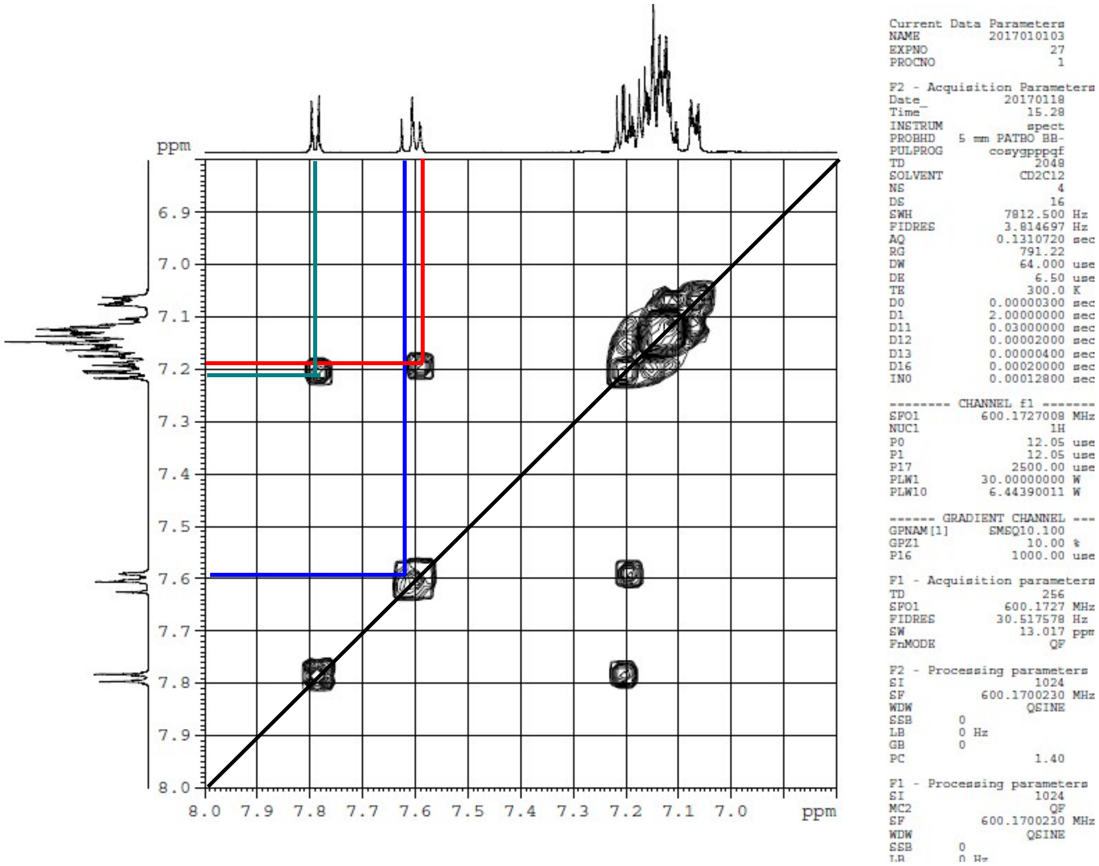

Figure S25. COSY NMR spectrum of compound **FBT-2TPE**. **(**aromatic region)

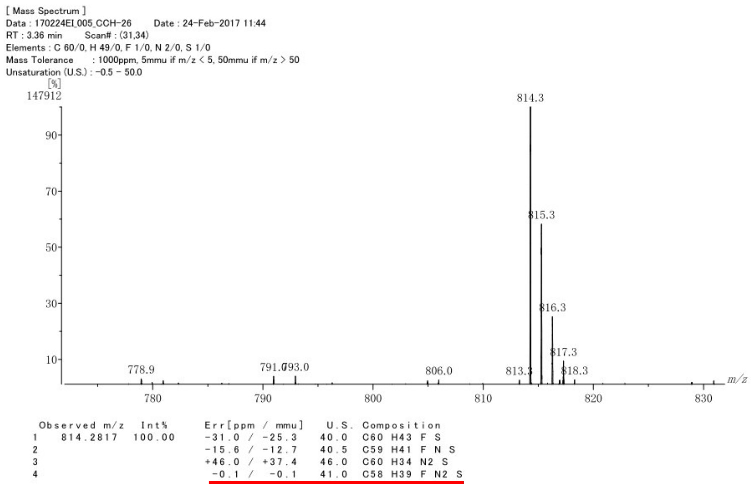

Figure S26. HR-EI Mass spectrum of compound **FBT-2TPE**.

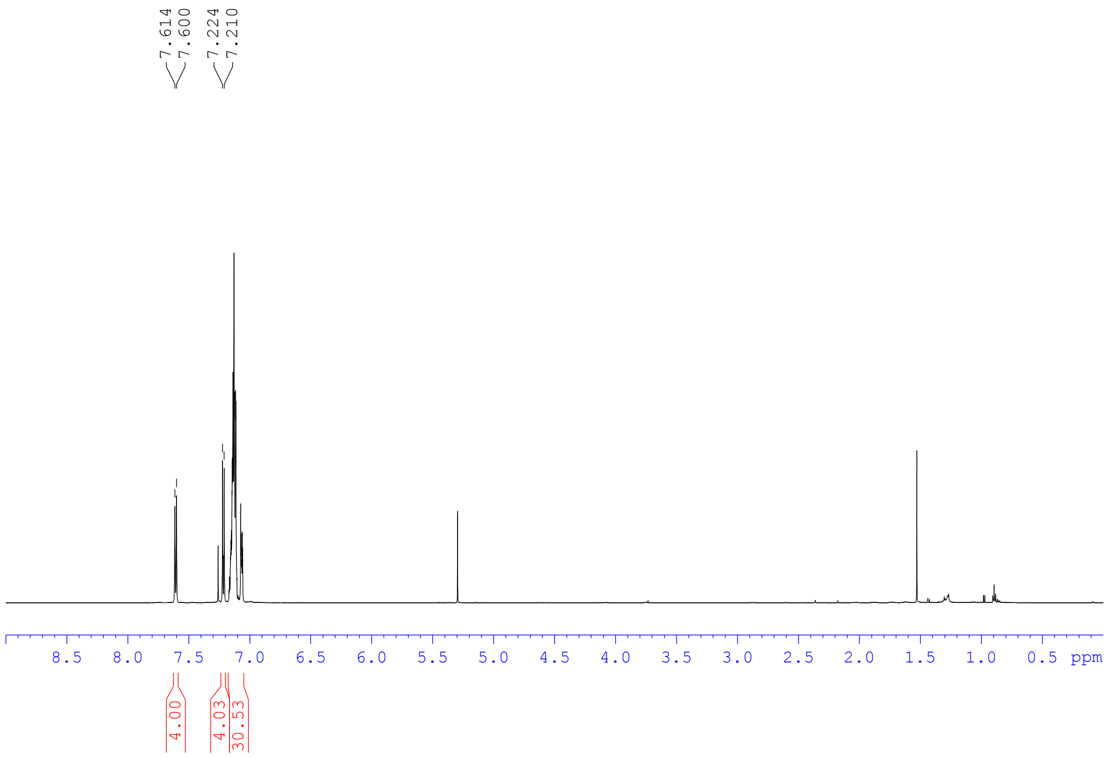

Figure S27. ^{1}H NMR spectrum of compound **2FBT-2TPE**.

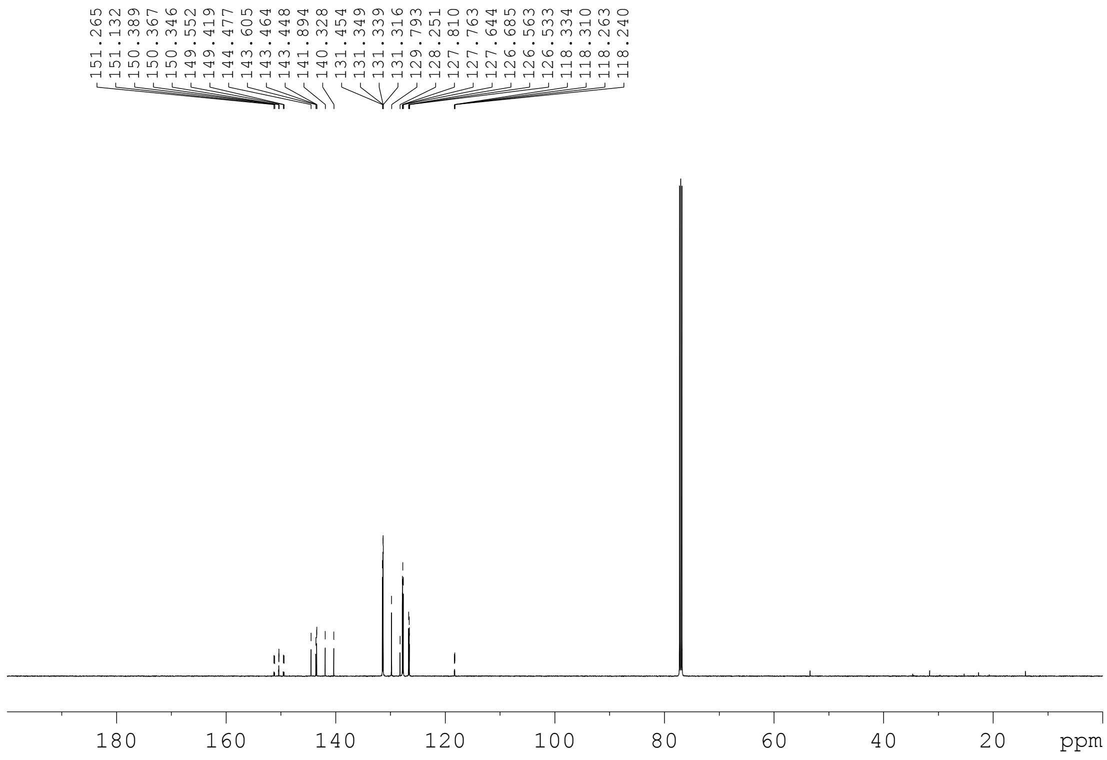

Figure S28. ^{13}C NMR spectrum of compound **2FBT-2TPE**.

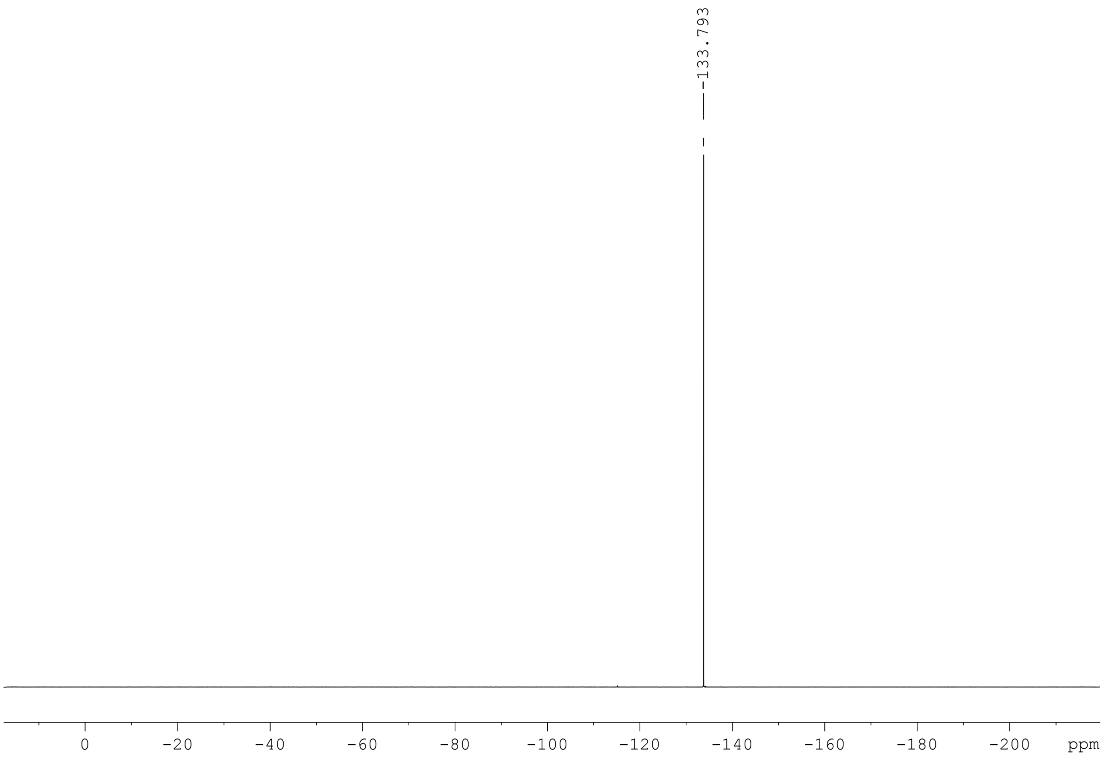

Figure S29. ^{19}F NMR spectrum of compound **FBT-2TPE**.

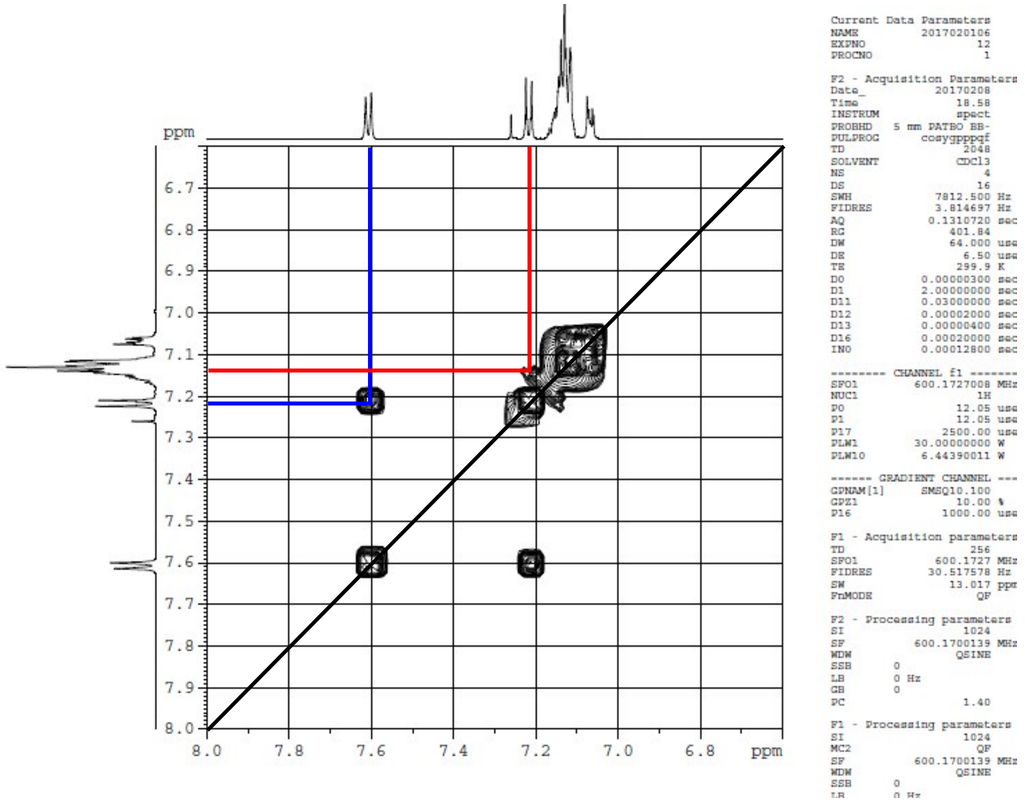

Figure S30. COSY NMR spectrum of compound **2FBT-2TPE**.

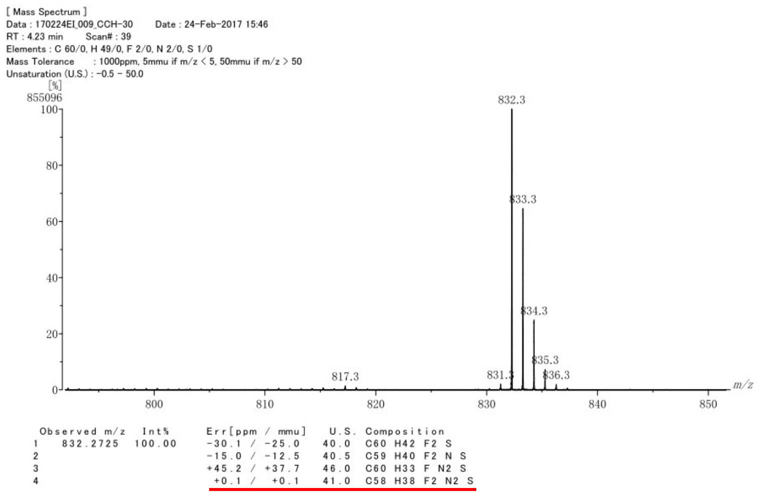

Figure S31. HR-EI Mass spectrum of compound **2FBT-2TPE**.

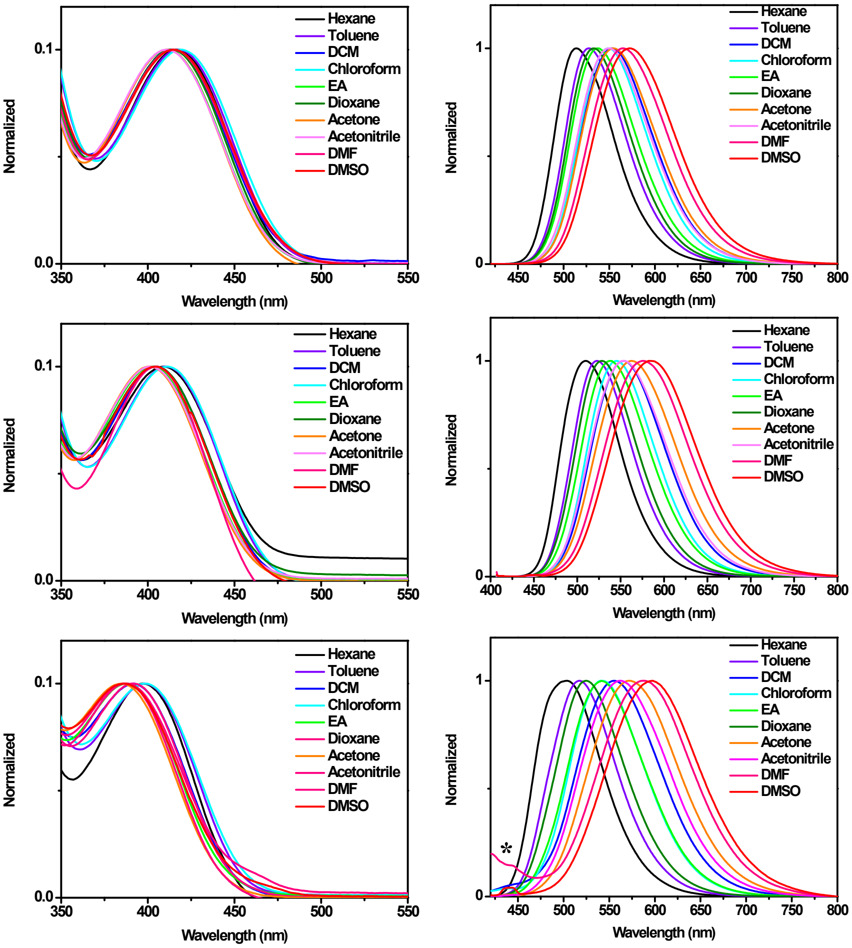

Figure S32. Normalized UV-Vis absorption spectra (left) and PL emission spectra (right) of **BT-2TPE** (top), **FBT-2TPE** (middle) and **2FBT-2TPE** (bottom) in different solvents.

Table S1. Solution and solid state absorption and emission maximum, quantum yield, Stokes shift and optical band gap.

	Solution			Film		E_g-opt (eV)	Stocks shift
	λ_{abs}	λ_{em}	QY	λ_{abs}	λ_{em}	film	Solution/film
**BT-2TPE**	414	553	0.204	444	535	2.35	139/91
**FBT-2TPE**	405	554	0.148	429	528	2.40	149/99
**2FBT-2TPE**	391	556	0.071	417	522	2.46	165/105

Table S2. Quantum yield with different water fraction in THF/water mixture.

	Quantum yield									
	0%	10%	20%	30%	40%	50%	60%	70%	80%	90%
**BT-2TPE**	0.239	0.222	0.205	0.181	0.171	0.142	0.107	0.212	0.242	0.233
**FBT-2TPE**	0.231	0.172	0.139	0.110	0.092	0.068	0.049	0.237	0.297	0.294
**2FBT-2TPE**	0.232	0.085	0.061	0.047	0.036	0.025	0.025	0.188	0.268	0.307

Table S3. UV-Vis absorption and emission maximum, quantum yield and optical bandgap data in various solvents.

	**BT-2TPE**			**FBT-2TPE**			**2FBT-2TPE**		
	λ_{abs}	λ_{em}	$ø_F$	λ_{abs}	λ_{em}	$ø_F$	λ_{abs}	λ_{em}	$ø_F$
Hexane	416	514	0.372	409	510	0.351	397	503	0.460
Toluene	418	528	0.327	411	523	0.293	399	517	0.190
DCM	414	553	0.204	405	554	0.147	391	556	0.071
Chloroform	418	549	0.236	411	545	0.200	400	542	0.159
EA	411	531	0.263	402	538	0.193	388	542	0.108
Dioxane	414	533	0.331	405	528	0.269	397	525	0.186
Acetone	411	554	0.182	401	563	0.100	385	571	0.058
Acetonitrile	411	550	0.192	401	553	0.115	387	562	0.068
DMF	415	565	0.126	404	576	0.070	388	587	0.027
DMSO	415	573	0.113	404	584	0.051	386	597	0.017

Table S4. Crystal data and structure refinement for **2FBT-2TPE**.

Identification code	d19031	
Empirical formula	C60 H42 Cl4 F2 N2 S	
Formula weight	1002.82	
Temperature	200(2) K	
Wavelength	0.71073 Å	
Crystal system	Triclinic	
Space group	P -1	
Unit cell dimensions	a = 9.1294(12) Å	α= 93.116(4)°.
	b = 9.2735(13) Å	β= 97.317(4)°.
	c = 16.422(2) Å	γ= 115.044(4)°.
Volume	1240.2(3) $Å^3$	
Z	1	
Density (calculated)	1.343 Mg/m^3	
Absorption coefficient	0.331 mm^{-1}	
F(000)	518	
Crystal size	0.69 x 0.45 x 0.11 mm^3	
Theta range for data collection	2.50 to 25.04°.	
Index ranges	-10<=h<=10, -11<=k<=11, -19<=l<=19	
Reflections collected	32746	
Independent reflections	4314 [R(int) = 0.0585]	
Completeness to theta = 25.04°	98.7 %	
Absorption correction	multi-scan	
Max. and min. transmission	0.9645 and 0.8039	
Refinement method	Full-matrix least-squares on F^2	
Data / restraints / parameters	4314 / 0 / 334	
Goodness-of-fit on F^2	1.035	
Final R indices [I>2sigma(I)]	R1 = 0.0447, wR2 = 0.1104	
R indices (all data)	R1 = 0.0489, wR2 = 0.1143	
Largest diff. peak and hole	0.435 and -0.530 e.$Å^{-3}$	
